# Lipid-based nanosystem of edaravone: development, optimization, characterization and *in vitro/in vivo* evaluation

**DOI:** 10.1080/10717544.2017.1337825

**Published:** 2017-06-21

**Authors:** Ankit Parikh, Krishna Kathawala, Chun Chuan Tan, Sanjay Garg, Xin-Fu Zhou

**Affiliations:** School of Pharmacy and Medical Sciences, Sansom Institute for Health Research, Division of Health Sciences, University of South Australia, Adelaide, Australia

**Keywords:** Edaravone, glucuronidation, permeability, oral bioavailability, lipid-based nanosystem

## Abstract

Edaravone (EDR) is a well-recognized lipophilic free radical scavenger for diseases including neurodegenerative disease, cardiovascular disease, and cancer. However, its oral use is restricted due to poor oral bioavailability (BA). The aim of present research was to enable its oral use by developing a lipid-based nanosystem (LNS). The components of LNS including oil, surfactants, and co-surfactants were selected based on their potential to maximize the solubilization in gastrointestinal (GI) fluids, reduce its glucuronidation and improve transmembrane permeability. The liquid LNS (L-LNS) with Capryol™ PGMC (Oil), Cremophor^®^ RH 40:Labrasol^®^:TPGS 1000 (1:0.8:0.2) (Surfactant) and Transcutol P^®^ (Co-surfactant) were optimized to form microemulsion having droplet size (16.25 nm), polydispersity index (0.039), % Transmittance (99.85%), and self-emulsification time (32 s). It significantly improved the EDR loading as well as its metabolism and permeability profile during transport across the GI tract. To overcome the possible drawbacks of L-LNS, Aerosil^®^ 200 was used to formulate solid LNS (S-LNS), and its concentration was optimized based on flow properties. S-LNS possessed all quality attributes of L-LNS confirmed by solid-state characterization, reconstitution ability, and stability study. The dissolution rate of EDR was significantly enhanced with L-LNS and S-LNS in simulated gastric, and intestinal fluids. The pharmacokinetic study revealed significant improvement in relative BA, *C*_max_, and *t*_1/2_ with L-LNS and S-LNS against EDR suspension. Moreover, S-LNS showed superior cellular uptake and neuroprotective effect compared to EDR in SH-SY5Y695 cell line. An appropriate selection of the components of LNS could enable effective oral delivery of challenging therapeutics that are conventionally used by the parenteral administration.

## Introduction

Free radicals contribute significantly to the pathogenesis of a variety of diseases associated with the organs including brain, heart, lung, liver, pancreas, intestine, and kidney (Valko et al., 2007; Lien Ai Pham-Huy et al., 2008; Kikuchi et al., 2012). Modulators of free radicals showed promising therapeutic efficacy in *in vitro* and *in vivo* studies but found limited success in clinical trials attributable to achieving a subtherapeutic level at the target site (Rahman, 2007; Kikuchi et al., 2011). Edaravone (EDR), a free radicle scavenger (3-methyl-1-phenyl-2-pyrazolin-5-one, MCI 186), showed its ability to diffuse into the most organs including brain, removed hydrogen radicals and displayed protective effects (Kikuchi et al., 2011, 2012). Radicut^®^, a parenteral preparation developed and marketed by Mitsubishi Tanabe Pharma Corporation, established global attention by receiving approval as a first neuroprotective drug in Japan for the treatment of acute ischemic stroke (Lapchak, 2010). Additionally, EDR showed significant pharmacological efficacy in the incurable diseases like Amyotrophic lateral sclerosis and Alzheimer diseases (Yoshino & Kimura, 2006; Jiao et al., 2015). Furthermore, the secret formula for oral administration was developed by Treeway, a biotechnology company from Netherlands. It has been accomplished with an orphan designation for Amyotrophic lateral sclerosis by regulatory agencies including European Medicines Agency (2014) (Anonymous, 2015b) and U.S. Food and Drug Administration (USFDA) (2015) (Anonymous, 2015a).

A liquid solution for intravenous infusion is the only EDR preparation available in the current market (Lapchak, 2010). It’s approved dosage regimen for acute ischemic stroke is one ampoule (30 mg of EDR) diluted with physiological saline and administered intravenously over 30 min twice a day for up to 14 days (Sinha et al., 2009). There is no oral formulation of EDR commercially available even though its therapeutic efficacy against diseases like Alzheimer and Cerebral aneurysm reported via oral administration in animals (Hudson et al., 2013, 2015). The oral route is the preferred route of administration for patients with chronic neurodegenerative diseases and doctors due to the ease of administration, flexibility in dose, improved quality of life, reduced the need for the hospital stay and low cost of treatment (Bala et al., 2016). Poor oral bioavailability (BA) could be a major challenge in enabling its oral use.

Edaravone is a lipophilic molecule having the low molecular weight of 174.2 g/mol with pka value of 7 (Borges et al., 2012). It belongs to biopharmaceutics classification system class IV drug due to poor solubility (1.85 mg/mL) and permeability (*P*_eff_ = 3.18 ± 0.0706 * 10^−7 ^cm/s) (Rong et al., 2014). EDR is substrate for P-glycoprotein (Pgp) efflux pump and uridine 5′-diphospho-glucuronosyl-transferase (UGT) enzymes, respectively (Ma et al., 2012; Rong et al., 2014). The poor oral BA (*F*_abs_ 5.23) could be attributed to poor aqueous solubility, stability, permeability across the gastrointestinal (GI) tract and extensive glucuronidation (phase II metabolism). Several investigations based on complexation with (2-hydroxypropyl)-β-cyclodextrin were reported to improve dissolution and permeability of EDR (Jian Zenga et al., 2010; Rong et al., 2014). However, these investigational findings have not effectively translated to use for the clinical purpose. A novel oral delivery system of EDR based on co-solvency and pH modification technology showed 5.71 fold enhancement of oral BA by improving solubility, metabolism, and permeability (Parikh et al., 2016). To take additional advantage of superior drug loading, facilitates transport through the intestinal lymphatic system, allowing enterocyte-based transport to enhance drug uptake, efflux, and deposition; lipid-based nanosystem (LNS) of EDR was considered to develop (Ali Khan et al., 2013; Sandeep Kalepu et al., 2013).

Lipid-based nanosystem is based on a strategy called ‘lipid-based nanoformulations’ specifically self-microemulsifying drug delivery system (SMEDDS). SMEDDS has been successfully utilized to enhance oral BA of drugs such as puerarin (Yi et al., 2017), tectorigenin (Zhang et al., 2017), exemestane (Singh et al., 2009), coenzyme Q10 (Balakrishnan et al., 2009), curcumin (Grill et al., 2014), and Fenofibrate (Kim et al., 2013). It is an isotropic mixture of oil, surfactant, and co-surfactants, which forms microemulsion having droplet size <100 nm after dilution with gentle stirring. Improvement of oral BA with lipid-based nanoformulations strategy is be owing to either individual or a combination of various factors, including greater solubilization, stabilization, and modifying permeability and metabolism profile (Dixit et al., 2010). By producing nanosized droplets, it facilitates rapid dissolution and absorption (Yeom et al., 2015). Moreover, it also protects the drug from GI environment as almost 100% drug could be entrapped in the microemulsion (Wu et al., 2015). Also, due to the presence of oil, nano-sized droplets could diffuse via lymphatic circulation which bypasses first pass metabolism (Dokania & Joshi, 2015). The selection of surfactants is the critical step as it plays a significant role in modifying metabolism and permeability profile by inhibiting cytochrome P450, UGT enzymes and modulating Pgp efflux pump (Hoosain et al., 2015; Zhou et al., 2015; Zhang et al., 2016). Sandimmune, Neoral, Norvir, and Fortovase are commercially successful products based on lipid-based nanoformulations strategy (Wais et al., 2016).

The commercial success of liquid lipid-based nanoformulations is limited due to stability concern including the interaction of components with gelatin capsules, the interaction between the components and/or issues related to oxidation of the oil (Bi et al., 2016). By converting liquid to solid lipid-based nanoformulations using solid adsorbents, it is possible to overcome issues including stability and handling problems. Various hydrophilic carriers including lactose, sorbitol, mannitol and hydrophobic carriers including colloidal silica, dextran; were widely utilized (Oh et al., 2011; Yeom et al., 2016). To the best of our knowledge, there is no lipid-based nanoformulations-based strategy applied to enhance oral BA of EDR.

In the present research, LNS was assessed to enable effective oral delivery of EDR. The initial screening of surfactants was conducted based on the ability of inhibition of EDR glucuronidation. Solubility and emulsification studies were performed to finalize oil, surfactant, and co-surfactant for the development of liquid LNS (L-LNS). Ternary phase diagram was constructed to optimize the concentration of each component of L-LNS based on criteria such as droplet size (<100 nm), polydispersity index (PDI <0.1), % Transmittance (>99%) and self-emulsification time (<120 s). *In-vitro* characterization of L-LNS including drug loading, determination of drug content, thermodynamic stability, and its reconstitution ability in GI fluids. Moreover, its *in vitro* effect on metabolism and permeability of EDR across the everted sacs of rat gut were evaluated. Solid LNS (S-LNS) was developed with Aerosil^®^ 200 and further optimized based on its flow properties. Also, differential scanning calorimetry (DSC), X-ray diffraction (XRD), scanning electron microscopy (SEM), transmission electron microscopy (TEM) and dissolution studies were conducted for further characterization. The stability of the S-LNS formulation was evaluated in various simulated GI fluids and after dilution to different folds. Additionally, an *in vivo* performance of L-LNS and S-LNS was studied with pharmacokinetic study in rats against EDR suspension.

## Materials and methods

### Materials

Edaravone was obtained from Aladdin Industrial Corporation (Shanghai, China). Capryol^™^ PGMC, Labrasol^®^, Transcutol P^®^, Capryol^™^ 90, Peceol^™^, Labrafil^®^ M1944cs, Lauroglycol^™^ FCC, Labrafac^™^ PG, Labrafil^®^ M2115, Lauroglycol^™^ 90, Labrafac^™^ Lipophile WL 1349 were received as gratis samples from Trapeze Associates Pvt Ltd., a representative of Gattefosse in Australia and New Zealand (Victoria, Australia). The samples of Captex^®^ 355, Captex^®^ 300, Capmul^®^ MCM C8, Capmul^®^ MCM EP, Capmul^®^ PG 8, Capmul^®^ PG 12 were received from Abitec Corporation (Janesville, WI). Acetic acid, phosphoric acid, sucrose, magnesium chloride, sodium hydroxide pellets, glycerol, polysorbate 20, and polysorbate 80 were purchased from Chem Supply (South Australia, Australia). Corn oil, cotton seed oil, oleic acid, N-methyl-2-pyrrolidone (NMP), propylene glycol, poly (ethylene glycol) 300 (PEG 300), PEG 400, 1,3-propanediol, potassium pyrophosphate, Tyrode’s solution, ascorbic acid, sodium carboxymethylcellulose, DMSO, concentrated HCL, di-sodium hydrogen phosphate, citric acid, perchloric acid, formic acid, uridine diphosphate glucuronic acid trisodium salt (UDPGA), alamethicin, and d-glucaric acid 1,4-lactone were purchased from Sigma-Aldrich (New South Wales, Australia). Cremophor^®^ RH 40 and Cremophor^®^ EL were obtained from BASF (Ludwigshafen, Germany), TPGS 1000 from Connell Bros Company Australasia Pvt Ltd. (Victoria, Australia), Miglyol^®^ 812 from IOI Oleo GmbH (Hamburg, Germany), peanut oil was purchased from PCCA (Houston, TX); sunflower oil from Goodman Fielder (New South Wales, Australia); castor oil from Wille Laboratory (Queensland, Australia); Triethanolamine from BDH Chemicals (Victoria, Australia); Caprylic triglyceride from Goldschmidt Chem Corporation (Victoria, Australia); Aerosil^®^ 200 from Evonik Industries AG (Hanau, Germany), Ethanol from Ajax fine chem (New South Wales, Australia), and Pierce bicinchoninic acid assay (BCA) Protein Assay Kit from Thermo Fisher Scientific (Victoria, Australia). Dulbecco’s modified Eagle’s medium (DMEM), Fetal bovine serum (FBS), penicillin, streptomycin and l-glutamine was purchased from Life Technology (Victoria, Australia). Water was purified by a MilliQ water purification system (Millipore Ultra-Pure Water System; Millipore, Australia). All the HPLC grade mobile phase components were procured from Merck (Victoria, Australia).

### Formulation development of L-LNS formulation

#### Selection of oil

The selection of oil was carried out by saturated solubility study. An excess amount of EDR was added to the different vehicles (1 ml of each) in glass vials and shaken continuously on a mechanical shaker (Model: so4036, Axyos Technologies, Brisbane, Australia) for 24 h at room temperature. The resultant mixtures were centrifuged (Model: Centrifuge 5415 R, Eppendorf) at 13,000 revolutions per minute (RPM) for 10 min. The supernatant was further diluted with methanol or petroleum ether and mobile phase. The determination of EDR solubility was analyzed by previously reported ultraviolet-visible (UV) spectrophotometers detector – high-performance liquid chromatography (HPLC) method after suitable dilution with methanol or petroleum ether and mobile phase (Parikh et al., 2016).

#### Selection of surfactant

The selection of surfactant was performed in three steps including *In vitro* glucuronidation assay (step 1), solubility (as mentioned in the selection of oil) (step 2), and emulsification ability (step 3).

##### *In vitro* glucuronidation assay

*In vitro* glucuronidation assay was carried out to examine the influence of different surfactants on the metabolism of EDR as described previously (Parikh et al., 2016). The differential centrifugation method was used to prepare rat liver microsomes, and its concentration was determined using BCA protein assay kit as per the supplier’s instruction. The level of EDR and EDR glucuronide (EDR-G) metabolite were determined with established extraction and analytical liquid chromatography-tandem mass spectrometry (LC/MS/MS) method (Parikh et al., 2016).

The mass spectrophotometric analysis was carried out by using a Quadrupole LC/MS/MS (Shimadzu, Kyoto, Japan) API 3000 system equipped with electrospray ionization and negative mode as discussed previously (Parikh et al., 2016). The mixture of methanol:water (50:50) samples were used to reconstitute the samples. The LC isolation was performed with Phenomenex Luna C18 (50 mm × 3 mm × 3 μm) column. The samples were gradient eluted with mobile phase A (MPA) (5% methanol + 95% water + 0.1% formic acid) and B (MPB) (95% methanol + 5% water + 0.1% formic acid) at the flow rate of 0.2 ml/min with total run time of 10 min. The gradient program was: 0–7.5 min, 30% MPA and 70% MPB; 7.5–8 min, 100% MPB, 8–10 min, 85% MPA and 15% MPB. The injection volume was 15 μl. Phenazone was used as an internal standard. The specific transition for EDR and Phenazone were *m/z* 175.1→  *m/z* 133.1 and *m/z* 189.1 → *m/z* 147.1, respectively.

##### Emulsification study

Screening of the selected surfactants was finalized based on their emulsification ability (Jain et al., 2014; Yeom et al., 2015). A 0.5 g of Capryol™ PGMC was mixed with the same amount of different surfactants and vortexed for about 2 min. The resultant mixture was warmed nearly 50 °C for 1 min. The final mixture (500 mg) was then diluted with water (500 mL) to obtain a dispersion with gentle shaking. All the mixtures were kept for 2 h to equilibrate and evaluated by measuring droplet size, and PDI using Malvern Zetasizer Nano ZS (Model ZEN3600), and % transmittance using UV–Visible Spectrophotometers (Thermo Scientific™ Evolution™ 201) at 638 nm. The standard protocol for measurement of droplet size and PDI after dilution was established by referring to the literature (Jain et al., 2014). The samples were collected at different time points after dilution. The significant decrease in droplet size and PDI were observed after 60 and 120 min of equilibrium compared to the time 0 min. The droplet size and PDI were remain stable after 120 min (Figure S1). Thus, 120 min (2 h) was considered as a standard time for equilibrium before measuring the droplet size and PDI in all studies. The emulsification time was also used as a parameter to determine the self-emulsification ability of the respective surfactants.

#### Selection of co-surfactant

The selection of co-surfactant was carried out in two steps including from solubility (as mentioned in the selection of oil) (step 1) and emulsification study (step 2).

##### Emulsification study

The selection of co-surfactant was finalized based on their emulsification ability (Jain et al., 2014). A 0.3 g of the selected surfactant was mixed with 0.2 g of different co-surfactants followed by an addition of 0.5 g of the oily phase. Droplet size, PDI, % transmittance and self-emulsification time were observed.

#### Construction of ternary phase diagrams

To identify the self-emulsification region, a ternary phase diagram was plotted for the different ratios of an oil Capryol™ PGMC, surfactants Cremophor^®^ RH 40:Labrasol^®^:TPGS 1000 in the ratio of 1:0.8:0.2, and a co-surfactant Transcutol P^®^ using Chemix^®^ School Software, trial version 3.6 (Oslo, Norway) (Dangre et al., 2016). In this mixture, the concentration of an oil, surfactant, and co-surfactant was varied from 0% to70% (w/w), 30% to 80% (w/w) and 0% to 30% (w/w), respectively. Initially, the concentration of oil, surfactant, and co-surfactant was altered in the order of 10%, and selection criteria were droplet size (<100 nm) and PDI (<0.1) (Dixit et al., 2010; Kanaujia et al., 2014). Later, the selected range of concentration oil, surfactant, and co-surfactant were further altered in the order of 5% and selection criteria were as kept as follow: droplet size (<100 nm), PDI (<0.1), % transmittance (>99%) and self-emulsification time (<120 s) (Rao et al., 2011; Jain et al., 2014). This particular region was considered as a self-microemulsifiying region in the plotted pseudo ternary phase diagram. The selection of L-LNS was conducted based on formulation with maximum oil concentration and minimum surfactant concentration and ability to produce microemulsion with having quality attributes mentioned above after dilution.

#### *In vitro* characterization of L-LNS

##### Globule size, PDI, and zeta potential

About 500 mg of L-LNS was diluted with 500 ml of water and mixed by gentle hand shaking to obtain a homogenous dispersion (Borhade et al., 2008). Droplet size, zeta potential, and PDI of the resultant microemulsion was measured using Malvern Zetasizer.

##### Percentage of transmission test and self-emulsification time

About 900 mg of the L-LNS was added drop by drop to the dissolution flask containing 900 ml of required aqueous media in United States pharmacopeia (USP) paddle type II Sotax Dissolution Apparatus (Victoria, Australia) dissolution apparatus at 37 °C and 75 RPM (Wei et al., 2005). % Transmittance against water and self-emulsification time were determined using UV–visible spectrophotometers at 638 nm and visual observation, respectively.

##### Drug loading ability

The maximum drug loading of EDR was determined based on the solubility protocol described in the selection of oil section. The stability of L-LNS formulation was visually observed for 24 h.

##### Determination of drug content

About 500 mg of L-LNS was diluted with methanol and vortexed for 15 min to extract the drug completely. The samples were then centrifuged for 10 min at 13,000 RPM. The supernatant was filtered through 0.45-μm polyvinyl difluoride syringe filters and further diluted with a suitable amount of mobile phase to analyze with previously developed UV-HPLC method.

##### Cloud point measurement

The cloud point is one of the important parameters to determine the reliability of the microemulsion-based formulation (Zhang et al., 2008). 500 mg of the L-LNS was diluted with 500 ml distilled water and placed in a water bath (Ratek Instruments, Adelab scientific, South Australia, Australia) with a slow rise in temperature. The cloud point was visually observed at the point where the solution becomes cloudy.

##### Thermodynamic stability study

The aim of this study was to check the stability of the L-LNS against temperature and centrifugal force (Singh et al., 2008). The test was performed in three steps including heating–cooling cycle, centrifugation, and a freeze–thaw cycle.

Step 1 (Heating cooling cycle): L-LNS was gone through six heating–cooling cycles of storage at each temperature of 4 °C and 45 °C, for not less than 48 h and observed for precipitation.

Step 2 (centrifugation): The formulation was further evaluated by centrifugation at 5000 RPM for 30 min and evaluated for phase separation.

Step 3 (freeze–thaw cycle): In the last step of the freeze–thaw cycle, the formulation was stored for 48 h at each temperature −10 °C and 25 °C. The formulation was observed visually for precipitation, creaming, and cracking.

##### Reconstitution behavior

The reconstitution study of the L-LNS was carried out in water, simulated gastric fluid (SGF, pH 1.2) and simulated intestinal fluid (SIF, pH 6.8) (Jain et al., 2014). The SGF and SIF were prepared as previously reported (Park et al., 2003). Briefly, 500 mg of L-LNS was dispersed in 500 ml of aqueous media. The reconstituted behavior of L-LNS was evaluated by measuring droplet size, PDI and emulsification time.

##### *In vitro* evaluation of L-LNS composition on metabolism and permeability of EDR

*In vitro* permeation and metabolism study was carried out as per our previous study (Parikh et al., 2016). Everted sacs of rat gut were prepared from male Wistar rats (180–200 g) to conduct the assay. Each sac was filled with two mL of Tyrode’s solution having EDR concentration of (50 μg/ml) in EDR suspension in 0.5% sodium carboxymethyl cellulose, with borneol (200 μg/ml) and L-LNS. The amount of EDR and EDR-G and the rate of permeation were determined.

### Formulation development of solid LNS formulation

#### Preparation of S-LNS

The EDR loaded S-LNS was adsorbed on Aerosil^®^ 200 in a different ratio of L-LNS to Aerosil^®^ 200 (1:0.4, 1:0.6, 1:0.8, and 1:1) by physical mixing using a mortar and pestle (Laddha et al., 2014). The mixture was passed through 150 μm sieve to get uniformity and dried at room temperature. The resultant mixtures were evaluated for flow properties (Laddha et al., 2014; Yeom et al., 2016). The optimum concentration of Aerosil^®^ 200 was selected based on the standards mentioned in USP 35 (Table S1) (Anonymous, 2012).

#### Optimization of concentration of solid carriers

Flow characteristics of the S-LNS formulations were determined to select the best concentration of solid carrier based on different parameters including the Carr’s index (CI), Hausner’s ratio (HR) and angle of repose (AR).

##### CI and HR

The cylinder method was utilized to calculate the tapped density and bulk density. An accurately weighed S-LNS was filled into the measuring cylinder, and the apparent volume was noted to determine the bulk density. The measuring cylinder was tapped for 100 times, and then the reduced volume was noted to calculate tapped density. The CI and HR were calculated by using the following Equations (1) and (2).

(1) $$\text{CI }\left(\%\right)=\frac{\text{Tapped}\;\text{density}-\text{Bulk}\;\text{density}}{\text{Tapped}\;\text{density}}*100$$

(2) $$\text{HR}=\frac{\text{Tapped}\;\text{density}}{\text{Bulk}\;\text{density}}$$

##### AR

The static funnel method was used to determine the AR of S-LNS. In brief, the sample was poured from the top of the funnel until the top of the pile touched to the tip at a particular height of 1.5 cm on a flat horizontal surface. The AR was calculated by using the formula, tan *θ* = height (*h*)/radius (*r*) where *r* is the radius of a pile of the powder.

##### *In vitro* characterization of S-LNS formulation

The determination of droplet size, PDI, drug content, % Transmittance and self-emulsification time were performed as described in a characterization of S-LNS section (Jain et al., 2014).

##### Solid state characterization

The solid state characterization of EDR, Aerosil^®^ 200 and S-LNS formulation was investigated by DSC, XRD, and SEM.

Differential scanning calorimetry study was conducted to evaluate the thermal characteristic of EDR, Aerosil^®^ 200 as a solid carrier, and the S-LNS (Cerpnjak et al., 2015). The DSC was carried out by using TA Instruments Discovery DSC (Model 2920). The samples (2–4 mg) were placed and sealed in hermetic aluminum pans. The measurement was executed over the temperature range from room temperature to 300 °C at a heating rate of 10 °C/min under nitrogen gas (50 ml/min).

The PXRD patterns of EDR, Aerosil^®^ 200 and the S-LNS, were recorded by using an X-ray diffraction instrument (PANalytical, Empyrean X-ray diffractometer) (Lim et al., 2015). X-ray diffraction patterns were acquired using CuKα radiation (*λ* = 1.5418 Å) on an X-ray diffractometer operating at 40 kV and 40 mA between 2 and 90° 2*θ* at a step size of 0.013° with a fixed 0.25° divergence slit, 0.50° anti-scatter slit and scanning rate of 2° min^−1^.

The external morphological characteristics of EDR, Aerosil^®^ 200, and S-LNS were studied by SEM (Lim et al., 2015). The samples were mounted on a SEM stub with conductive double-sided adhesive. The ultra-high resolution secondary electron microscopy (Zeiss Microscopy Merlin with GEMINI II column) was used to study the surface characteristics. The SEM equipped with a field emission gun was operated at 0.7 kV to acquire the secondary electron images.

##### TEM analysis upon reconstitution

The morphology of the microemulsion was analyzed using Cryo-TEM, wherein the copper grids were first dipped into a sample solution and immediately transferred into liquid nitrogen and allowed to stay in for 10 min (Kuntsche et al., 2011). The Copper grid was freeze dried and analyzed using transmission electron microscope using JEOL JEM-1010. S-LNS of EDR (500 mg) was dispersed in 500 ml of water to generate the microemulsion.

##### Stability in simulated GI fluids

The stability of formulation was carried out in water, SGF and SIF (Jain et al., 2014). 500 mg of S-LNS was dispersed into 500 ml of an aqueous media, incubated for 24 h further evaluated for droplet size, PDI, and drug precipitation.

##### Stability after dilution to different folds

Solid LNS was dispersed in the SGF at different folds (200, 400, 600, and 800) (Jain et al., 2014). The samples were further evaluated for droplet size, PDI, and drug precipitation.

### *In vitro* release test

The *in vitro* release was carried out with dialysis bag method by using USP dissolution apparatus type II (paddle) at 37 °C and 75 RPM speed (Kamboj & Rana, 2016). The EDR suspension, L-LNS and S-LNS formulation equivalent to 30 mg of EDR were filled in a dialysis bag (Cellu·Sep T4 12,000–14,000 molecular weight, Seguin, TX). The dialysis bag was sealed from both sides with clamps and placed in the dissolution flask containing 900 ml of dissolution media including SGF and SIF. Samples (5 mL) were collected at different time intervals of 5, 15, 30, 45, 60, 90, 120 min, respectively and replaced with fresh dissolution medium. The collected samples were further analyzed by previously developed UV-HPLC method after dilution with mobile phase. The release kinetic of EDR from suspension, L-LNS and S-LNS were determined by putting obtained data in various kinetic models including first order, Zero order, Hixson–Crowell, Higuchi-matrix, and Korsmeyer–Peppas (Costa & Sousa Lobo, 2001; Jaiswal et al., 2014; Kamboj & Rana, 2016). The relevant correction coefficient was considered to select the best model. Moreover, the dissolution profiles of EDR suspension, L-LNS, and S-LNS in SGF and SIF were compared using similarity factor (f2) and dissimilarity factor (f1) approach. All calculations were performed based on the equations mentioned in Tables S2 and S3. In general, f1 values 0–15 and f2 values 50–100 demonstration the similarity of the dissolution profiles (Costa & Sousa Lobo, 2001).

### Pharmacokinetic study

The approved protocol from the University of South Australia (Australia), was used as discussed previously using male Sprague–Dawley rats (300 ± 25 g) (Parikh et al., 2016). Three groups of rats (*n* = 6) were orally administered with EDR suspension, L-LNS, and S-LNS at an equivalent dose of 30 mg/kg. To extract EDR from the plasma matrix, a mixture of Mcllvaine buffer of pH 5.4 and dichloromethane-n-pentane (3:7 v/v) was used as described previously (Parikh et al., 2016). The EDR level was determined by previously developed LC/MS/MS method. The pharmacokinetic parameters were calculated using Phoenix WinNonlin software.

### *In vitro* neuroprotection assay

#### Cell culture

SH-5Y5YAPP695 human neuroblastoma cells were obtained from American Type Culture Collection (ATCC, Rockville, MD). The cells were allowed to grow in the DMEM media containing 10% FBS, 2 mM l-glutamine, and 50 IU/mL of each penicillin and streptomycin followed by incubating at 37 °C in a humidified incubator supplemented with 95% air and 5% CO_2_.

#### MTT assay

The cell viability assay was performed on SH-SY5YAPP695 cell lines to determine the protective effect of EDR and S-LNS on the cell growth against the cytotoxicity induced by CuSO_4_, H_2_O_2_ and Abeta 42. The cells were incubated with CuSO_4_ (0.5 μM,) H_2_O_2_ (50 μM) and Abeta 42 (1 μM). The EDR (3 μM) and S-LNS (equivalent to 3 μM) was also added at the same time with addition of cytotoxic agents. After 19 h of incubation, 25 μl of MTT (Sigma-Aldrich, St. Louis, MO, 5 mg/ml in phosphate-buffered saline) was added to the each well after removing the medium. Dimethyl sulfoxide (200 μL) was added to dissolve the insoluble purple formazan product to produce a colored solution. The optical density (OD) was read at 570 nm wavelength on the multi-well scanning spectrophotometer (WALLAC 1420 (PerkinElmer, Waltham, MA). All the experiments were performed in triplicate.

#### *In vitro* cellular uptake study

SH-SY5YAPP695 cell line was seeded at the density of 2 × 10^5^ cells/mL in 6-well plate (Invitrogen, Mulgrave, VIC, Australia) and incubated at 37 °C overnight. The cells were then replaced by the culture media containing EDR and S-LNS and incubated at 37 °C for 0.5 and 2 h. At the end of the incubation, the cells were washed two times with cold PBS followed by lysing in radioimmunoprecipitation assay buffer containing 50 mM Tris, 150 mM NaCl, 1 mM EDTA, 0.5% Triton X-100, 0.5% Sodium deoxycholate, pH 7.4, and protease inhibitors including 1 mM phenyl methane sulfonyl fluoride (PMSF), antipain, pepstatin, and leupeptin (Roche, Australia). The EDR concentration was determined using LC/MS/MS method against original amount to quantify the cellular uptake.

### Statistical analysis

GraphPad Prism 6 was used for statistical analysis. All values were indicated as mean ± standard deviation (SD). The statistical analysis of data was performed by using Student’s *t*-test for two groups, one-way and two-way analysis of variance (ANOVA) for multiple groups. The mean differences were considered significantly valued in all experiments at *P* < 0.01.

## Results and discussion

### Formulation development of L-LNS

#### Selection of oil

The selection of appropriate oil based on solubility study is critical to prevent precipitation of drug during storage and before undergoing in situ solubilization (Jain et al., 2014). The interference study of each excipient was conducted during the solubility study. After extracting EDR with either methanol or petroleum ether, no change was observed in shape of the peak and retention time of EDR which confirmed the absence of interference of excipient during estimation of EDR concentration using UV-HPLC method (Figure S2). Table 1 shows the solubility profile of EDR in several natural and synthetic oils. Capryol™ PGMC (34.63 ± 2.52 mg/mL) revealed the highest solubilization capacity among different oils followed by Capmul^®^ MCM EP (24.08 ± 1.24 mg/mL) and Labrafil^®^ M2125 (22.14 ± 1.51). The solubility of EDR was lower in natural oils compared to synthetic oils. The oils showed higher solubilization for EDR compared to water (1.85 mg/mL) might be owing to its lipophilic (log p: 1.2) nature (Parikh et al., 2016). In a case of the lipophilic drug, higher solubility in oils could increase drug loading and facilitates absorption through lymphatic route and therefore provide protection against the first pass metabolism (Gupta et al., 2013). Capryol™ PGMC is chemically propylene glycol monocaprylate having hydrophilic–lipophilic balance value (HLB) of 6. The components of hard and gelatin capsule are compatible with Capryol™ PGMC. It confirms to US Pharmacopeia – National Formulary standard for Human and Veterinary applications (Anonymous, 2010). It was successfully used to enhance the oral BA of drugs including Fenofibrate (Kim et al., 2013) and Flutamide (Verma et al., 2011). Capryol™ PGMC was selected as an oil for further development.

**Table 1.** Solubility of EDR in selected ingredients (mean ± S.D., *n* = 3).

Oil	Solubility (mg/mL)
Capryol™ PGMC	34.63 ± 2.52
Capryol™ 90	21.20 ± 1.45
Captex^®^ 355	9.61 ± 1.41
Peceol™	6.91 ± 1.56
Penanut oil	7.38 ± 0.97
Sunflower oil	8.83 ± 1.46
Miglyol^®^ 812	11.17 ± 2.92
Corn oil	6.31 ± 1.64
Caprylic triglyceride	8.10 ± 1.56
Cotton seed oil	3.37 ± 0.85
Castor oil	5.78 ± 1.63
Lauroglycol™ FCC	1.54 ± 0.32
Labrafac™ PG	1.77 ± 0.23
Captex^®^ 300	7.74 ± 1.35
Labrafil^®^ M2115	22.14 ± 1.57
Lauroglycol™ 90	18.02 ± 2.13
Labrafac™ Lipophile WL 1349	7.26 ± 1.52
Capmul^®^ MCM C8	22.11 ± 2.56
Capmul^®^ MCM EP	24.08 ± 1.24
Capmul^®^ PG 8	18.17 ± 1.76
Capmul^®^ PG 12	16.88 ± 2.36
Oleic acid	7.37 ± 2.52
Surfactant/solvents	
Labrasol^®^	62.48 ± 5.56
Labrasol^®^: TPGS 1000 (4:1)	58.63 ± 4.74
Cremophor^®^ RH 40	26.09 ± 3.11
Glycerol	9.92 ± 1.42
Polysorbate 20	14.65 ± 3.64
Cremophor^®^ EL	9.95 ± 1.86
Polysorbate 80	8.29 ± 3.56
Triethanolamine	19.42 ± 3.26
Co-surfactants/solvents	
Transcutol P^®^	93.06 ± 6.21
Propane diol	21.54 ± 2.26
Ethanol	70.28 ± 5.27
PEG 300	50.63 ± 4.63
Labrafil^®^ M1944cs	9.55 ± 1.36
NMP	37.79 ± 3.67
PEG 400	6.40 ± 1.47
Propylene glycol	5.93 ± 1.63

#### Selection of surfactant

The role of surfactant in lipid-based nanoformulations is the most critical in modulating pharmacokinetic profile of drugs as a solubilizer, stabilizer, for inhibition of enzymes including UGT and cytochrome p450 for metabolism of drugs; and as a permeability enhancer (Christiansen et al., 2011; Zhou et al., 2015). EDR is extensively metabolized and has an issue of poor permeability across GI tract due to substrates of UGT enzyme and Pgp (Rong et al., 2014; Parikh et al., 2016). Hence, the selection of appropriate surfactant for the development of L-LNS could consider three steps including *in vitro* glucuronidation assay, solubility, and emulsification ability test.

**Figure 1.**
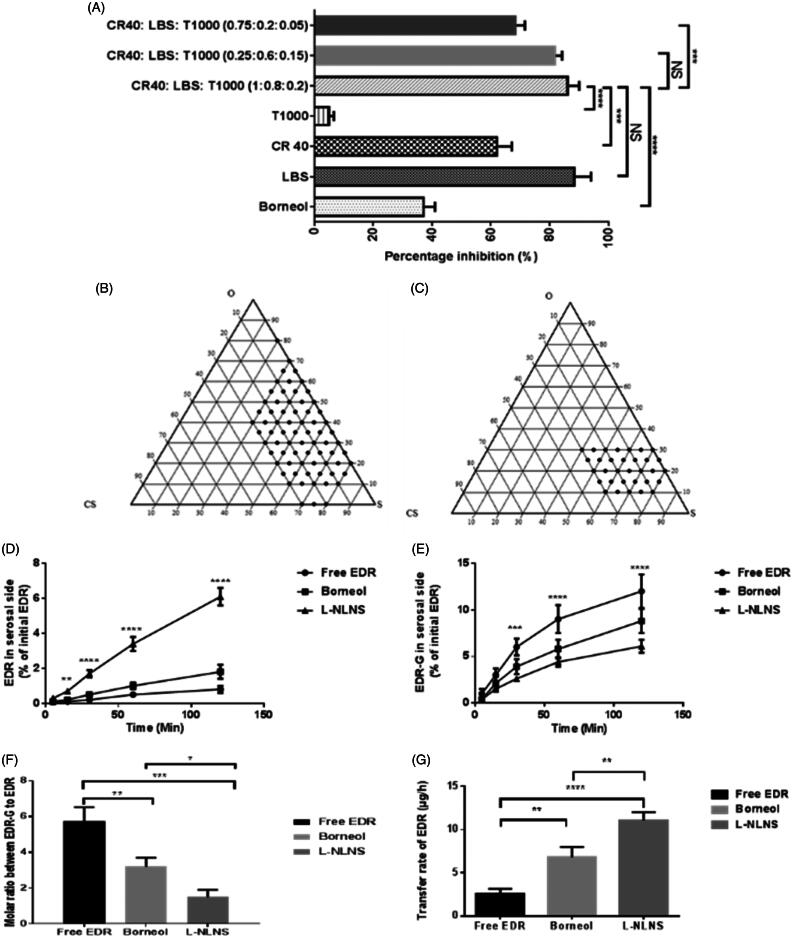
Inhibitory effect of various surfactants on glucuronidation of EDR in a microsomal incubation assay (A) ***P < 0.001 and ****P < 0.0001 for LBS: CR40: T1000 (1:0.8:0.2) compared to others. Data for Borneol and LBS were adopted from the earlier report (Parikh et al., 2016). LBS: Labrasol^®^, T1000: d-α-Tocopherol polyethylene glycol 1000 succinate, CR40: Cremophor^®^ RH 40 and NS: not significant (*p* > 0.05); Construction of ternary phase diagram with the system of Capryol^TM^ PGMC as oil, Cremophor^®^ RH 40: Labrasol^®^: TPGS 1000 (1:0.8:0.2) as a surfactant and Transcutol^®^ P as a co-surfactant explored self-emulsifying region (B), obtained self-emulsifying region (C); The effect of formulation ingredients of L-LNS on permeability and metabolism of EDR during transportation across everted sacs of rat small intestine. The amount in percentage of EDR (D) and EDR-G (E) with L-LNS compared to EDR suspension on serosal side of everted sac at various time interval, molar ratio between EDR-G and EDR (F), and transfer rate of EDR in the serosal side of rat everted gut sacs (G) (mean ± S.D., *n* = 3). *P < 0.05, **P < 0.01, ***P < 0.001 and ****P < 0.0001. Two-way or One-way ANOVA and Sidak's multiple comparisons test. Data for EDR suspension and Borneol were adopted from the earlier report (Parikh et al., 2016).

As a step 1, Labrasol^®^, Cremophor^®^ RH 40 and TPGS 1000 were selected from the literature which showed a significant effect on UGT and Pgp, thereby improves oral BA of various drugs (Prasad et al., 2003; Collnot et al., 2007; Zhou et al., 2015). The inhibitory effect on the glucuronidation of EDR was assessed *in vitro* for each surfactant with a well-established assay using rat liver microsomes. Labrasol^®^ and Cremophor^®^ RH 40 showed the superior inhibitory effect on EDR metabolism compared to Borneol (as a positive control) and TPGS 1000 which is good agreement with previous reports (Figure 1A) (Parikh et al., 2016).

To avoid the precipitation of drug during the storage and formulate stable L-LNS, the solubility of EDR with various surfactants was investigated as a step 2 (Jain et al., 2014; Yeom et al., 2015). Use of a combination of surfactants in the development of lipid-based nanoformulations is a popular approach for increasing solubility as well as dissolution (Ilem-Ozdemir et al., 2015; Ishak & Osman, 2015). The highest solubility of EDR was observed in Labrasol^®^ (62.48 mg/mL) followed by Cremophor^®^ RH 40 (26.09 mg/mL) among all surfactants as showed in Table 1. The surfactants play a critical role in the spontaneous formation of stable and transparent microemulsion (Droplet size <100 nm and PDI <0.2). The emulsification ability of surfactants as an individual or in combination were further studied with Capryol™ PGMC as an oil and evaluated based on the following criteria such as droplet size (<100 nm), PDI (near to zero), % T (near to 100%) and emulsification time (<2 min) (step 3). The results are shown in Table 2. Labrasol^®^ and Cremophor^®^ RH 40 as individual surfactants were passed the criteria for emulsification time but failed for others and unable to generate microemulsion with desired characteristics. TPGS 1000 as a stabilizer, showed improvement of emulsification ability of labrasol^®^ by decreasing droplet size and PDI, and increasing % Transmittance but was unable to match the criteria. The ratio of Labrasol^®^ to TPGS 1000 (0.8:0.2) was selected from the literature to improve intestinal absorption of vancomycin, and further assessed for *in vitro* glucuronidation assay, solubility, and emulsification ability test. Later, the ratio of Cremophor^®^ RH 40 and combination of Labrasol^®^ to TPGS 1000 (0.8:0.2) was optimized 1:1 among other tried ratios (0.25:0.6:0.15) and (0.75:0.2:0.05) based on emulsification ability test and *in vitro* glucuronidation assay. The combination of Cremophor^®^ RH 40, Labrasol^®^ and TPGS 1000 (1:0.8:0.2) successfully formed a microemulsion with desired characteristics with better inhibitory effect on EDR glucuronidation among other combinations. Labrasol^®^ was considered as a control as it showed superior results (Parikh et al., 2016). No significant difference was observed compared to labrasol^®^ in both studies (Figure 1(A) and Table 1).

**Table 2:** Assessment of emulsification ability of surfactants and co-surfactants (mean ± SD, *n* = 3).

Surfactants	Droplet size (nm)	PDI	% Transmittance	Self-emulsification time
Screening of surfactants				
Labrasol^®^	282.52 ± 21.56	0.432 ± 0.141	68.51 ± 5.11	<2 min
Labrasol^®^: TPGS 1000 (0.8:0.2)	198.41 ± 12.24	0.357 ± 0.092	79.41 ± 4.62	<2 min
Cremophor^®^ RH 40	92.34 ± 8.92	0.341 ± 0.094	94.62 ± 3.59	<2 min
Cremophor^®^ RH 40 : Labrasol^®^ : TPGS 1000 (0.25:0.75)	169.85 ± 10.48	0.303 ± 0.082	87.59 ± 2.98	<2 min
Cremophor^®^ RH 40 : Labrasol^®^ : TPGS 1000 (0.75:0.25)	78.24 ± 6.57	0.281 ± 0.071	97.61 ± 1.56	<2 min
Cremophor^®^ RH 40 : Labrasol^®^ : TPGS 1000 (1:0.8:0.2)	69.51 ± 3.41	0.260 ± 0.064	98.21 ± 0.24	<2 min
Co-surfactants	Droplet size (nm)	PDI	% Transmittance	Emulsification time
Screening of co-surfactants				
Transcutol P^®^	31.41 ± 2.51	0.174 ± 0.025	99.08 ± 0.09	<2 min
Ethanol	58.26 ± 6.78	0.414 ± 0.104	94.52 ± 0.94	<2 min
PEG 300	98.83 ± 9.35	0.552 ± 0.162	91.84 ± 1.68	<2 min

Cremophor^®^ RH 40 is categorized as a polyoxyethylene castor oil derivatives having a hydrophobic portion (glycerol polyethylene glycol oxystearate with fatty acid glycerol polyglycol esters) and hydrophilic constitute (polyethylene glycols and glycerol ethoxylate). It has HLB value of 14–16 and considered as a nontoxic and nonirritant for oral administration from acute (median lethal dose (LD_50_): >20 g/kg/day), subacute and chronic toxicity studies (Rowe et al., 2009). It was successfully utilized for the development of lipid-based nanoformulation for a number of drugs to enhance oral BA including ritonavir (Deshmukh & Kulkarni, 2014) and seocalcitol (Grove et al., 2006). Labrasol^®^ is chemically Caprylocaproyl macrogol-8 glycerides having HLB value of 12. It is a mixture of monoesters, diesters, and triesters of glycerol and monoesters and diesters of polyethylene glycols with a mean relative molecular mass between 200 and 400. It is also proven to a relatively a nontoxic and nonirritant with median lethal dose value >20 ml/kg/day (Rowe et al., 2009). Some lipid-based nanoformulations with Labrasol^®^ are reported to improve physicochemical characteristics and oral absorption of drugs such as Exemestane (Singh et al., 2009) and Coenzyme Q10 (Balakrishnan et al., 2009). TPGS 1000 is chemically vitamin E polyethylene glycol 1000 succinate having a mixture of monoesterified polyethylene glycol 1000, the diesterified polyethylene glycol 1000, free polyethylene glycol 1000, and free tocopherol. It has HLB value 13.2. It is recognized as safe (GRAS) listed and also included in the FDA inactive ingredients database (Rowe et al., 2009). The use of TPGS 1000 as a stabilizer was reported previously with lipid-based nanoformulations (Kanaujia et al., 2014; Bala et al., 2016). The combination of Labrasol^®^ and TPGS 1000 (1:0.25) was successfully improved intestinal absorption of vancomycin hydrochloride (VCM) (Prasad et al., 2003).

From inhibitory effect on EDR glucuronidation, solubility, emulsification, the potential of inhibitory effect on Pgp efflux and safety profile, a combination of Cremophor^®^ RH 40, Labrasol^®^ and TPGS 1000 (1:0.8:0.2) was selected as a surfactant.

#### Selection of co-surfactant

To make stable L-LNS and improve emulsification ability of selected surfactant, the selection of co-surfactant was carried out in two steps including solubility and emulsification study. The maximum saturated solubility of EDR was observed in Transcutol P^®^ (93.06 mg/mL) among all co-surfactants (Table 1). From solubility study, Transcutol P^®^, ethanol and PEG 300 were selected for emulsification study. The appropriate co-surfactant with surfactant lowers the interfacial tension and forms a steady layer at oil–water interface (Jain et al., 2014). The microemulsion with ethanol and PEG 300, did not show desired characteristics. The significant decrease in droplet size from 69.51 nm to 31.41 nm and in PDI from 0.260 to 0.174 was observed in the case of Transcutol P^®^. Previously, Transcutol P^®^ has been effectively utilized to improve emulsification of lipid-based nanoformulations with Labrasol^®^ as a surfactant (Cirri et al., 2007; Oh et al., 2011). The results confirmed the candidature of Transcutol P^®^ as a co-surfactant. Chemically, it is diethylene glycol monoethyl ether. Its safety is also proven in various acute, subchronic and chronic toxicity studies with LD_50_ value >5 g/kg/day (Rowe et al., 2009). From solubility, emulsification and proven safety profile, Transcutol P^®^ was selected as a co-surfactant.

#### Construction of ternary phase diagrams

From studies mentioned above, Capryol™ PGMC was chosen as oil, Cremophor^®^ RH 40: Labrasol^®^: TPGS 1000 as a surfactant and Transcutol P^®^ as a co-surfactant for the development of L-LNS for EDR. It could spontaneously form an oil in water transparent microemulsion with gentle agitation after the dilution with aqueous media as it requires minimal requirement of free energy for emulsion formation (Singh et al., 2009). The optimization of the concentration of each component is critical to the formation of stable microemulsion after dilution. To identify a self-microemulsification region and to select the appropriate amount of selected oil, surfactant, and co-surfactant, Pseudoternary phase diagrams were constructed. The concentration of an oil 0–80% (w/w), surfactant 20–80% (w/w), and co-surfactant 0–30% (w/w) was tried. Initially, total 24 formulations were prepared and evaluated by altering the oil, surfactant and co-surfactant concentration in the order of 10%. The criteria for the selection of range for oil, surfactant, and co-surfactants for further screening, were based on droplet size (<100 nm) and PDI (<0.1). The measurement of droplet size was considered as a crucial factor as nanosize droplets influence the dissolution of the drug by providing a large surface area, thus enhance the drug absorption. It also affects the stability of microemulsion (Mohsin et al., 2016). The determination of PDI is used to understand the droplet size range in the system. The PDI value closer to zero indicates the uniformity in the size of droplets into the system. The PDI value of >0.3 indicates a heterogeneous dispersion while <0.1 designates homogenous dispersion (Kanaujia et al., 2014).

The ternary phase diagram (Figure 1B) was constructed based on the result of 24 trial formulations. Total 15 formulations with a composition of oil (10–40%), surfactant (40–80%) and co-surfactant (0–30%), were passed as per the acceptable criteria. In the absence of oil, the formulation made up with surfactant and co-surfactant were not able to form stable dispersion after dilution. A significant increase in the droplet size and PDI were witnessed with increasing concentration of an oil and decreasing concentration of surfactant and co-surfactant. The probable reasons for increasing the droplet size are an increment of bulk due to the high concentration of oil and an inability of reduction in interfacial tension at a lower concentration of surfactant (Laddha et al., 2014). The role of co-surfactant is critical with surfactant in forming a steady layer at the oil–water interface and preventing penetration of water in oil droplets. It could also play a significant role to solubilize the drug of required clinical dose (Jain et al., 2014).

The selected concentrations of oil (10–40%), surfactant (40–80%), and co-surfactant (0–30%) were further assessed by varying each concentration in the order of 5%. Total 46 formulations were tried to identify typical composition for L-LNS (Figure 1B). The criteria were set for the selection of typical composition for L-LNS such as maximum oil content with minimum surfactant content, and for microemulsion after dilution having minimum droplet size (<100 nm), minimum PDI (<0.1), % Transmittance (>99%) and self-emulsification time (<120 s) (Jain et al., 2014; Yeom et al., 2015). The concentration of oil is critical for a lipophilic drug like EDR as it could solubilize the drug and also facilitate the transport through lymphatic route and prevent its metabolism by protecting it from GI environment. It also affects the drug release and rate of absorption from GI tract. The concentration of surfactants was decided to keep minimum as its higher concentration could show unwanted toxicity and GI irritation (Kanaujia et al., 2014). Measurement of % Transmittance is the indicative of the clarity of microemulsion. The formulation with % Transmittance <99% compared to water, confirms the formation of a microemulsion containing droplets of nanosize (Kim et al., 2013). The determination of self-emulsification time is the assessment of the efficiency of emulsification after exposing to the aqueous media. The acceptable criteria of 120 s was set as per the previous literature (Kanaujia et al., 2014).

The droplet size of microemulsion was decreased with decreasing the concentration of oil and increasing the concentration of surfactant and co-surfactant. The higher amount of surfactant and co-surfactant lowers the interfacial tension, thus increasing the penetration of water to disrupt oil–water interface resulting in decreased droplet size. The range of droplet size of trial formulations was 15.4–129.3 nm. The PDI was in the range of 0.035–0.297 for all formulations. The higher proportion of oil in the formulation made the variable size of droplets and resulted in the higher PDI. The concentration of surfactant and co-surfactant played a significant role in stabilization of interfacial tension to generate a system having uniform droplet size and minimum PDI. The self-emulsification time was increased from 20 s to 120 s with increasing the concentration of oil in the composition and decreasing the concentration of surfactant and co-surfactant. The formulation with a high proportion of oil required more time for emulsification while the higher amount of surfactant and co-surfactant lowered the emulsification time. The increasing droplet size and/or PDI showed significant decreasing % Transmittance. The increasing concentration of oil and decreasing concentration of surfactant and co-surfactant varied % Transmittance in the range of 94.09–99.95. The self-microemulsifying region was plotted in Figure 1(C). Considering all the requirements, the L-LNS with Capryol^™^ PGMC as oil, the mixture of Cremophor^®^ RH 40, Labrasol^®^ and TPGS 1000 (1:0.8:0.2) as a surfactant and Transcutol P^®^ as a co-surfactant in the ratio of 30:25:25:20% w/w was finalized.

#### *In vitro* characterization of L-LNS formulation

##### Droplet size, PDI, and %transmittance

The optimized L-LNS showed 16.25 nm droplet size, 0.039 PDI and 99.85% of transmittance after dilution with water which confirms its potential to form a transparent uniform dispersion and to improve drug dissolution and absorption.

##### Self-emulsification time

The self-emulsification time for optimized L-LNS was found to be 32 s which suggests a requirement of very little amount of free energy for emulsification, thus spontaneous formation of a microemulsion of desired qualities.

##### Zeta potential

Zeta potential is the crucial parameter in determining the stability of microemulsion formed after dilution with aqueous media. The stability of formed microemulsion depends on the electrostatic force of droplets. A decreasing electrostatic repulsive force could destabilize the microemulsion and result in phase separation. Increasing the electrostatic forces will prevent aggregation of droplets and stabilize the microemulsion (Singh et al., 2009). The higher zeta potential compared to zero value indicates superior stability. L-LNS showed zeta potential of −9.37 millivolts which designates stable microemulsion.

##### Drug loading ability

The maximum loading ability was found 6.45% w/w. There was no precipitation observed before and after dilution with water during the storage for 24 h at room temperature.

##### Determination of drug content

The loading efficiency was determined to understand the actual amount of EDR loaded in L-LNS after preparation. L-LNS showed 99.74% of drug content which confirmed the uniform distribution of the drug in the formulation.

##### Cloud point measurement

The determination of the cloud point is the temperature at which irreversible phase separation, as well as dehydration of surfactants, results in the sudden appearance of cloudiness. Hence, it is critical to determine the integrity of microemulsion as a function of temperature (Zhang et al., 2008). The ideal cloud point should be more than 37 °C, a physiological temperature to avoid phase separation in physiological condition (Sallam & Marin Bosca, 2015). The cloud point of finalized L-LNS was found to be 72 °C suggesting its suitability for oral administration.

##### Thermodynamic stability study

The thermodynamic stability is the unique advantage of the microemulsion-based formulation over simple suspension and emulsion-based formulations (Sheikh Shafiq-un-Nabi et al., 2007). The thermodynamic stability of formulations was carried out to evaluate the effect of various stress conditions like temperature and centrifugal force. The optimized L-LNS was found thermodynamically stable with no phase separation, precipitation, creaming and cracking.

##### Reconstitution ability of L-LNS

After oral administration, L-LNS would encounter dilution with GI fluids like SGF and SIF. Therefore, the reconstitution ability of L-LNS was assessed by determining droplet size and PDI at various time intervals (Jain et al., 2014). There were no phase separation or drug precipitation and significant difference perceived in droplet size and PDI after dilution with water, SGF, and SIF up to 24 h (Table S4). The results revealed that the L-LNS is the robust after dilution with all aqueous media.

##### *In vitro* evaluation of L-LNS composition on metabolism and permeability of EDR

The extensive metabolism and poor permeability were considered as the major hurdle contributed to the poor oral BA of EDR (Rong et al., 2014; Parikh et al., 2016). To evaluate the effect of formulation ingredients of L-LNS on metabolism and transport from mucosal to serosal side, *in vitro* permeability and metabolism assay were conducted with everted sac of rat gut. Two-way ANOVA analysis by using Sidak’s multiple comparisons tests confirmed the significant effect of ingredients on metabolism as well as permeability. The L-LNS showed the significantly higher amount of EDR transfer after 15 min compared to EDR suspension and in combination with Borneol (Figure 1D). The amount of EDR transferred with L-LNS and Borneol were 7.63 fold (*p* < 0.0001) and 2.25 fold (*p* < 0.0001), respectively compared to EDR suspension after 120 min. EDR undergoes significant glucuronidation during the transport to the serosal side from mucosal. For EDR-G, the significantly small amount was observed on the serosal side with L-LNS compared to EDR suspension (1.96 fold) (*p* < 0.0001) and with Borneol (1.44 fold) (*p* < 0.001), which confirmed the significant effect on metabolism (Figure 1E). The molar ratio of EDR-G to EDR was found significantly low with L-LNS compared to EDR suspension (3.82 fold) (*p* < 0.001) and with Borneol (2.13 fold) (*p* < 0.05) (Figure 1F). Figure 1(G) shows the significant higher transfer rate (4.32 fold) of EDR with L-LNS compared to EDR suspension (4.32 fold) (*p* < 0.0001) and with Borneol (1.63 fold) (*p* < 0.001). The results confirmed the potential of L-LNS formulation for EDR to enhance the oral BA.

### Formulation development and characterization of S-LNS formulation

#### Selection and optimization of concentration of solid carriers

The possible drawbacks related to liquid lipid-based nanoformulation are poor stability, interaction with capsule material and low portability during the manufacturing. Solid lipid-based nanoformulation could offer the superior stability and patient compliance. Therefore various water-soluble carriers such as mannitol and lactose, and water-insoluble carriers including silicon dioxide were used previously to convert liquid to solid lipid-based nanoformulation (Bi et al., 2016). The water-insoluble carrier was preferred over water-soluble carriers because of possible inadequacy related to water-soluble carriers such as significantly low oil absorbing capacity and being relatively hygroscopic in nature which tends to recrystallize during the storage and deteriorate the quality features of liquid lipid-based nanoformulation (Yeom et al., 2016). Aerosil^®^ 200 was chosen on account of its small droplet size (<10 μm), larger surface area (>200 m^2^/g) and higher oil adsorbing capacity (255 mL/100 g) (Yeom et al., 2016). Additionally, it is widely used for pharmaceutical preparation for the oral and topical administration because of its proven safety profile with GRAS listing (Rowe et al., 2009).

The optimization of the concentration of Aerosil^®^ 200 was performed by evaluating the flow characteristics of the S- LNS (Laddha et al., 2014; Yeom et al., 2016). The CI, HR, and AR were considered as evaluation parameters and standards as per USP to categories the flow of S-LNS from very very poor to excellent (Anonymous, 2012). The result of various S-LNS based on a different ratio of L-LNS to Aerosil^®^ 200 is shown in Table S5. The flow of S-LNS improved with increasing the concentration of Aerosil^®^ 200 as CI, HI and AR were decreasing. With the ratio of L-LNS to Aerosil^®^ 200 (1:1), S-LNS displayed excellent flow as per USP standard with CI (9.67), HR (1.11) and AR (27.19). Therefore it has been selected for further characterization.

#### *In vitro* characterization of S-LNS formulation

##### Droplet size, PDI, %transmittance, and self-emulsification time

The characterization of S-LNS was evaluated to assess the quality of microemulsion formation diluting with water. S-LNS retained 98.56% of EDR content and previously mentioned all quality attributes after dilution like droplet size (75.25 ± 4.57 nm), PDI (0.07 ± 0.01), % Transmittance (99.48 ± 0.14), and self-emulsification time (38 ± 6).

##### Solid state characterization

**Figure 2.**
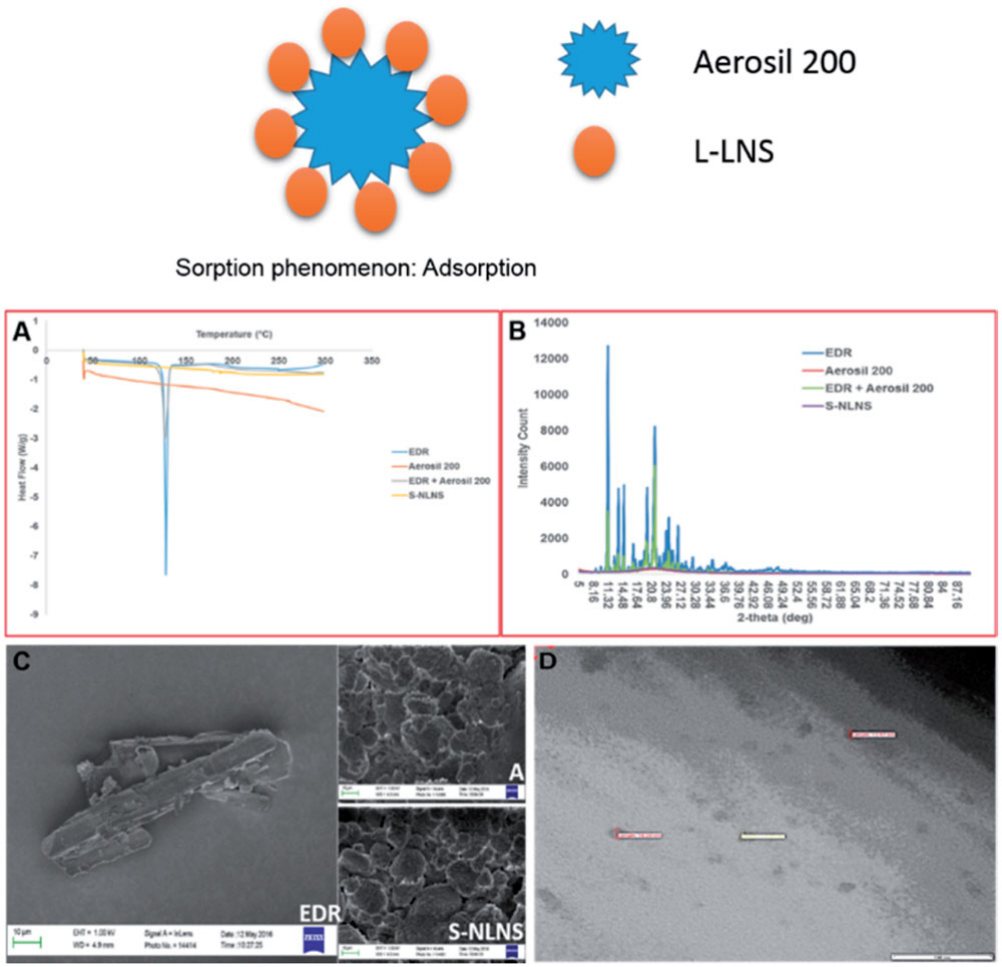
Graphical representation of sorption phenomena. Overlay of the DSC patterns (A), and PXRD patterns (B) of EDR, Aerosil^®^ 200, Physical mixture of EDR and Aerosil^®^ 200 and S-LNS. SEM images (C) of EDR, Aerosil^®^ 200 and S-LNS. TEM image (D) of microemulsion formed after dilution of S-LNS in water. Data of DSC, PXRD, and SEM for EDR were adopted from the earlier report. EDR: Edaravone and A: Aerosil 200.

The determination of the physical state of EDR in S-LNS was considered as it could influence its performance *in vitro* and *in vivo* (Yeom et al., 2016). Moreover, as Aerosil 200 is water insoluble carrier, L-NLNS should be adsorbed to the surface of Aerosil 200 (Yeom et al., 2016). The graphical representation of sorption phenomena is added in Figure 2. DSC, XRD and SEM analysis were performed for solid state characterization of S-LNS compared to EDR and Aerosil^®^ 200.

The DSC thermograms of pure EDR, Aerosil^®^ 200, a physical mixture of EDR and Aerosil^®^ 200 and S-LNS are revealed in Figure 2(A). A sharp endothermic peak in case of EDR at 128.39 °C was indicated its highly crystalline nature. No endothermic peak was observed with Aerosil^®^ 200, which could be due to amorphous nature. DSC curve for a physical mixture of EDR and Aerosil^®^ 200 displayed a small endothermic peak of EDR compared to the pure drug, which could be as a result of dilution with Aerosil^®^ 200. Interestingly, no endothermic peak of EDR appeared in the DSC spectra of S-LNS. It might be explained as EDR could exist in a solubilized and/or amorphous form in S-LNS compositions (Krstic et al., 2015).

The internal physical state of EDR in the S-LNS was further verified by XRD analysis. The XRD spectra of pure EDR, Aerosil^®^ 200, a physical mixture of EDR and Aerosil^®^ 200 and S-LNS are shown in Figure 2(B). The crystalline nature of EDR was reflected with several sharp and intense peaks. The low intense peaks of EDR were observed with a physical mixture of EDR, and Aerosil^®^ 200 might be as a result of dilution with solid carriers. Aerosil^®^ 200 did not show any peak because of amorphous in nature. The result of S-LNS showed the absence of distinct peaks of EDR confirming the complete solubilization of EDR in the composition of S-LNS, the thorough conversion in an amorphous state from crystalline state or disordered, crystalline state in the S-LNS (Yeom et al., 2016).

The analysis of surface morphology by SEM images of pure EDR, Aerosil^®^ 200 and S-LNS are shown in Figure 2(C). The rod-shaped structure of EDR confirmed the crystalline structure. The SEM image of Aerosil^®^ 200 displayed aggregates of amorphous particles with a porous surface. Distinct crystalline particles of EDR were not observed in the case of S-LNS suggesting complete adsorption of L-LNS into the solid carriers (Yeom et al., 2016).

##### TEM analysis

The morphology of microemulsion after reconstituting of S-LNS with water was examined with a transmission electron microscope (Figure 2D). The spherical shape, size (<100 nm) and Gaussian distribution were confirmed with TEM photography. The TEM results are consistent with the analysis of zeta sizer.

##### Stability in simulated GI fluids

The robustness of S-LNS was confirmed as similar to L-LNS with no significant difference in droplet size and PDI after dilution up to 24 h (Table S6). S-LNS revealed nearly five-fold, and 1.4–1.75 fold more droplet size and PDI compared to L-LNS, respectively after dilution with various aqueous media. An incomplete desorption of different components of S-LNS on water-insoluble carriers could be likely reason for increasing droplet size and PDI and consistent with the previous report (Yeom et al., 2016).

##### Stability after dilution to different folds

After an oral administration, S-LNS undergoes variable dilution with GI fluids. Thus, the stability of microemulsion was determined after diluting with various dilutions of SGF (Jain et al., 2014). There is no significant difference in droplet size and PDI of microemulsion formed from different dilution up to 24 h as shown in the Table S4. Additionally, no phase separation or drug precipitation was observed. All the quality attributes of L-LNS were retained in S-LNS after all dilutions.

### *In vitro* release test

**Figure 3.**
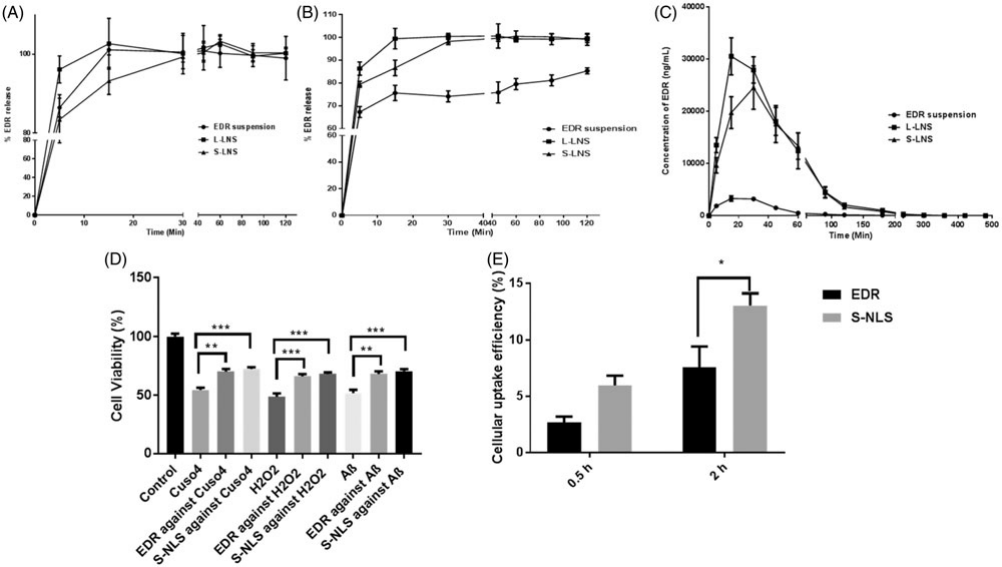
*In vitro* release profile of EDR suspension, L-LNS, and S-LNS in various dissolution media SGF (A) and SIF (B) (mean ± S.D., *n* = 3). The release rate of the L-LNS and S-LNS compared to EDR suspension were statistically significant (*p* < 0.0001) at all the time points in the SIF media while no significant difference (*p* > 0.05) was observed in the SGF media; Plasma concentration–time curves for the EDR suspension, L-LNS and S-LNS (30 mg/kg) in Sprague–Dawley rats after oral administrations (C) (mean ± S.D., *n* = 6). L-LNS and S-LNS showed statistically significant (*p* < 0.001) improvement of EDR’s plasma profile as a function of time up to 90 min compared to EDR suspension. Effect of EDR and S-LNS on cell viability in presence of CuSO_4_, H_2_O_2_ and Abeta 42 (D). *In vitro* cellular uptake efficiency of S-LNS and EDR SH-SY5Y695 cell line after incubating for 0.5 and 2 h (E) (mean ± S.E., *n* = 3). *P < 0.05, **P < 0.01, ***P < 0.001 and ****P < 0.0001. One and two-way ANOVA and Sidak's multiple comparisons test. Data for EDR suspension was adopted from the earlier report (Parikh et al., 2016).

After exposing formulation to the GI environment, the drug could be present in a free molecule, emulsion and/or micellar form. The standard dissolution testing could not mimic the *in vivo* dissolution as it might not separate the free drug molecule to the entrapped drug molecule in emulsion droplet or micelles form. The additional dialysis bag method was utilized to compare the *in vitro* release of EDR from EDR suspension, L-LNS, and S-LNS in SGF and SIF (Kamboj & Rana, 2016). The determination of equilibrium solubility of EDR in SGF and SIF was performed to check the possibility of a pH-dependent release of EDR as the pH-dependent solubility of EDR was reported from pH 2 to 10 (Parikh et al., 2016). Surprisingly, the superior solubility of EDR in SGF (8.26 ± 1.42 mg/mL) was observed compared to SIF (1.89 ± 0.51 mg/mL). The higher solubility in SGF could be explained due to the formation of salt as SGF is made up of hydrochloric acid which has a tendency to make salt with EDR. The results of EDR release from suspension, L-LNS, and S-LNS in SGF and SIF media are shown in Figure 3(A, B), respectively and analyzed by two-way ANOVA analysis using Sidak's multiple comparisons tests. In SGF, the EDR suspension demonstrated 86.45% and 100% drug release within 5 min and 15 min, respectively due to high solubility in SGF. The L-LNS showed significant improvement in dissolution (*p* < 0.05) with 96.14% EDR release within 5 min followed by 100% in 15 min, whereas S-LNS showed 83.42% EDR release in 5 min, 93.24% in 15 min and almost 100% in 30 min. There was a significant difference in dissolution profile of L-LNS and S-LNS observed at 5 min (*p* < 0.001) and 15 min (*p* < 0.05). In SIF, there was 85% drug release witnessed within 120 min with EDR suspension while 100% drug release within 15 and 30 min in case of L-LNS and S-LNS, respectively. The dissolution of EDR was significantly enhanced with L-LNS and S-LNS against EDR suspension at each time points (*p* < 0.0001). The novel approach overcomes the hindrance to the drug release of EDR related to characteristics of dissolution media. It could improve the dissolution by providing higher surface area because of generating nano-sized droplets and increasing the solubilization of EDR. There was the significant difference in dissolution profile in SGF and SIF of L-LNS, and S-LNS observed at 5 min (*p* < 0.001) and 15 min (*p* < 0.05). The possible reason could be hydrogen bonding between a silanol group and EDR (Yeom et al., 2016).

The result of release kinetic of EDR from suspension, L-LNS, and S-LNS are presented in Table S7. From suspension, the release kinetic of EDR follows Hixson–Crowell in SGF and Korsmeyer–Peppas model (following an anomalous transport with non-Fickian model) in SIF. It shows dissimilar release profile in SGF an SIF which confirmed the pH-dependent release (f2 value 27and f1 values 22). In a case of L-LNS, EDR release fits in Hixson–Crowell model and finds similar (f2 value 69 and f1 values below 3) in SGF and SIF. The EDR release with S-LNS follows the first order in SGF and SIF and similar release profile (f2 value 74 and f1 values below 2). The EDR release from suspension in SIF obeyed non-Fickian diffusion which suggests that the wetting of EDR particles by aqueous phase at the interface could play a vital role in its release. The Hixson–Crowell model described that the release rate of EDR from EDR suspension and L-LNS in SGF could not limit by the diffusion but on the dissolution rate of EDR particle. The EDR release from S- LNS followed first order kinetics which indicates that release would be depended on the proportional of the amount of EDR left in the interior of S-LNS in both SGF and SIF (Costa & Sousa Lobo, 2001). Also, L-LNS and S-LNS show similar release profile in SGF and SIF which confirms the pH-independent release of EDR in various dissolution media. The dramatic improvement in dissolution profile of EDR could play a critical role in the enhancement of its oral BA.

### Pharmacokinetic study

The pharmacokinetic study of L-LNS and S-LNS were performed to evaluate the effect of formulation composition on oral absorption against EDR suspension. The 30 mg/kg oral dose of EDR was selected from the literature and based on our previous publication which showed its pharmacological effect against Alzheimer disease (Hayashi et al., 2003; Jiao et al., 2015; Nakajima et al., 2015). The plasma concentration-time profile and pharmacokinetic parameters estimated by using Phoenix WinNonlin software were showed with the comparison of the *p* value in Figure 3(C) and Table 3, respectively. Two-way ANOVA analysis by using Sidak's multiple comparisons tests was performed to analyze the improvement statistically between the groups. There is a significant enhancement of *C*_max_ and Area under the curve (AUC) with L-LNS (9.27 and 10.78 fold) and S-LNS (7.45 and 9.29 fold) than EDR suspension. S-LNS revealed longer *T*_max_ whereas no significant difference was witnessed in EDR suspension and L-LNS. L-LNS and S-LNS presented a marked improvement in *t*_1/2_ value compared to EDR suspension. Most importantly, the relative BA of L-LNS and S-LNS were 1079% and 929% compared with EDR suspension, respectively. The relative BA, *C*_max,_ and AUC of S-LNS were slightly lower than the L-LNS. The possibility of incomplete desorption of L-LNS on Aerosil^®^ 200 could be a potential reason for a slightly low *C*_max_ and AUC value of S-LNS than S-LNS. S-LNS displayed longer *t*_1/2_ value compared to L-LNS as the slow release of liquid components from solid carriers showed in dissolution study could be responsible. The hydrogen bonding interaction between a silanol group of Aerosil^®^ 200 and EDR could be another potential factor (Yeom et al., 2016).

**Table 3:** Pharmacokinetic parameters obtained by Phoenix WinNonlin software (mean ± SD, *n* = 6).

Parameters	EDR suspension	L-NLNS	S-NLNS	*p* Value^a^	*p* Value^b^	*p* Value^c^
*C*_max_ (ng/mL)	3290.42 ± 507.41	30525.13 ± 2014.5	24521.32 ± 3451.69	<0.0001	<0.0001	NS
*T*_max_ (min)	15.19 ± 1.45	12.52 ± 2.03	30.56 ± 5.29	NS	<0.01	<0.01
*t*_1/2_ (min)	58.31 ± 3.52	78.49 ± 2.53	85.12 ± 3.81	<0.001	<0.001	NS
AUC_0-t_ (ng·min/mL)	164,185 ± 15,264	1,770,750 ± 854,244	1,524,862 ± 624,240	<0.0001	<0.0001	NS
*F*_0–t_ (%)	100	1078.5 ± 155.9	928.75 ± 140.89	<0.0001	<0.0001	NS

Unpaired Student’s *t*-test for two groups. Data for EDR suspension was adopted from the earlier report (Parikh et al., 2016).

NS: on-significant.

^a^Comparison between EDR suspension and L-NLNS.

^b^Comparison between EDR suspension and S-NLNS.

^c^Comparison between L-NLNS and S-NLNS.

In our previous and current study, the crystalline nature of EDR was confirmed with DSC, XRD and SEM study (Parikh et al., 2016). The selected dose for the pharmacokinetic study was 30 mg/kg for EDR suspension, L-LNS, and S-LNS. The solubility of EDR in suspension was 1.89 mg/mL so additional EDR would be present in undissolved suspension form. In L-LNS and S-LNS, the dose of EDR was completely dissolved due to solubilization effect of the components of the new system. Therefore, enhancement of solubility and dissolution would be potential reasons for enhancement of BA. The significant impact of its composition on metabolism and permeability of EDR as shown in the *in vitro* assay could play a critical role in the improvement of oral BA of EDR. The possible higher cellular uptake with microemulsion system could also contribute to the improvement of intestinal absorption (Bala et al., 2016). Cremophor^®^ RH 40, Labrasol^®^ and TPGS 1000 have an ability to modulate Pgp efflux pump (Prasad et al., 2003; Collnot et al., 2007; Zhou et al., 2015). Therefore, their combination could significantly inhibit its excretion through Pgp efflux and enhance EDR absorption. For EDR, modulation of Pgp efflux pump and inhibition on UGT enzyme are the critical requirements (Rong et al., 2014; Parikh et al., 2016). Thus, L-LNS and S-LNS with a combination of Cremophor^®^ RH 40, Labrasol^®^ and TPGS 1000, showed significant improvement in oral BA of EDR.

### *In vitro* neuroprotection assay

To study the neuroprotective effect of EDR and S-LNS, a human derived neuroblastoma cell line SH-SY5Y was used which is a common model cell line to test the neurotoxicity *in vitro* (Mi et al., 2012). The effective dose of EDR (3 μM) was discovered from our pervious study using SH-SY5Y695 cell line (Jiao et al., 2015). Hydrogen peroxide is a physiological constituent of living cells and is continuously produced via diverse cellular pathways. Moreover, cytotoxicity induced by hydrogen peroxide, copper metal ions and Abeta 42 play critical role in AD pathogenesis and contribute for reduction in neuron viability (Atwood et al., 2004; Milton, 2004; Mayes et al., 2014). The strong neuroprotective action of EDR and S-LNS was observed against the cytotoxicity induced by CuSO_4_, H_2_O_2_ and Abeta 42 (Figure 3(D)). S-LNS showed greater but not statistically significant neuroprotective effect which could be due to the protective effect of TPGS (Mi et al., 2012). Additionally, higher cellular uptake of S-LNS was observed compared to EDR after incubating for 0.5 h and 2 h (Figure 3(E)). The higher cellular uptake and better neuroprotective effect of LNS make LNS as a promising and very safe therapeutic candidate for the further development at preclinical and clinical stage.

## Conclusions

The present study reports the development of EDR loaded LNS to enable its efficient oral delivery by enhancing the oral BA. The selection of excipients including oil, surfactants, and co-surfactant were based on their potential to improve the physicochemical parameters of EDR including solubility, stability, and metabolism. L-LNS containing Capryol^™^ PGMC (30%), Cremophor^®^ RH 40: Labrasol^®^: TPGS 1000 (1:0.8:0.2) (50%) and Transcutol P^®^ (20%) significantly inhibited the metabolism and enhanced permeability across the rat gut. The S-LNS formulated by using Aerosil^®^ 200, not only retained all quality attributes including droplet size, PDI, % Transmittance and self-emulsification time of L-LNS formulation after dispersed in aqueous media, but also showed superior *in vitro* dissolution compared to EDR suspension. The L-LNS and S-LNS showed excellent potential for further development of liquid and solid dosage form by enhancing 10.79 fold and 9.29-fold oral BA of EDR, respectively. Additionally, S-LNS showed higher cellular uptake and better neuroprotective effect compared to EDR in SH-SY5Y695 cell line. The use of appropriate ingredients for the LNS is demonstrated to enable effective oral delivery of EDR like challenging therapeutics that are conventionally dosed by injection.

## Supplementary Material

IDRD_Sanjay_et_al_Supplemental_Content.pptx
