# Supplementary material for: Lipid-based nanosystem of edaravone: development, optimization, characterization and in vitro/in vivo evaluation
Source: Drug Deliv. 2017 Jun 21;24(1):962–78. doi: 10.1080/10717544.2017.1337825 (PMC8241028; doi:10.1080/10717544.2017.1337825)
Supplement: IDRD_Sanjay_et_al_Supplemental_Content.pptx [file IDRD_A_1337825_SM7745.pptx]

## Slide 1
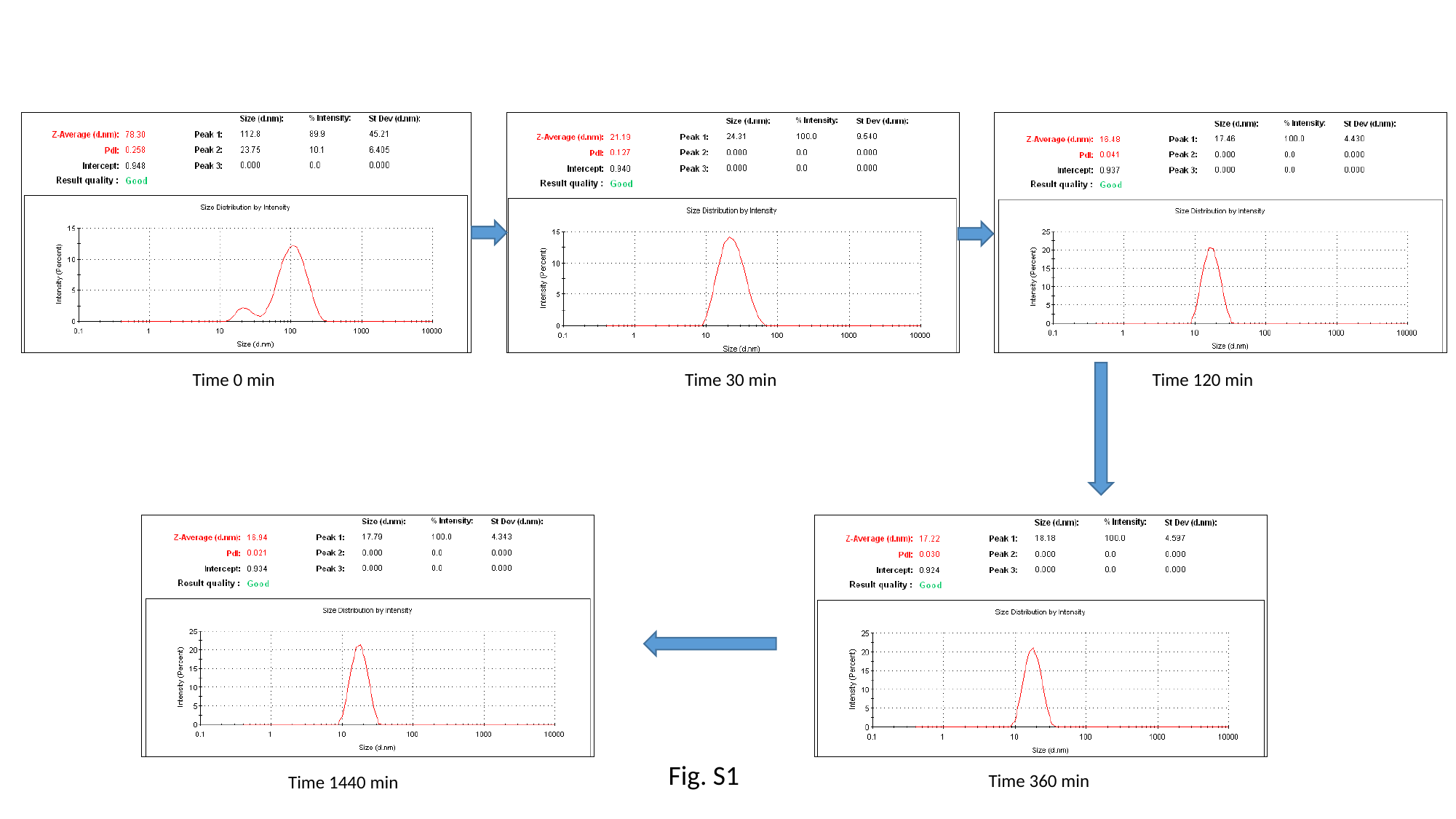

Time 0 min
Time 30 min
Time 120 min
Fig. S1
Time 360 min
Time 1440 min

## Slide 2
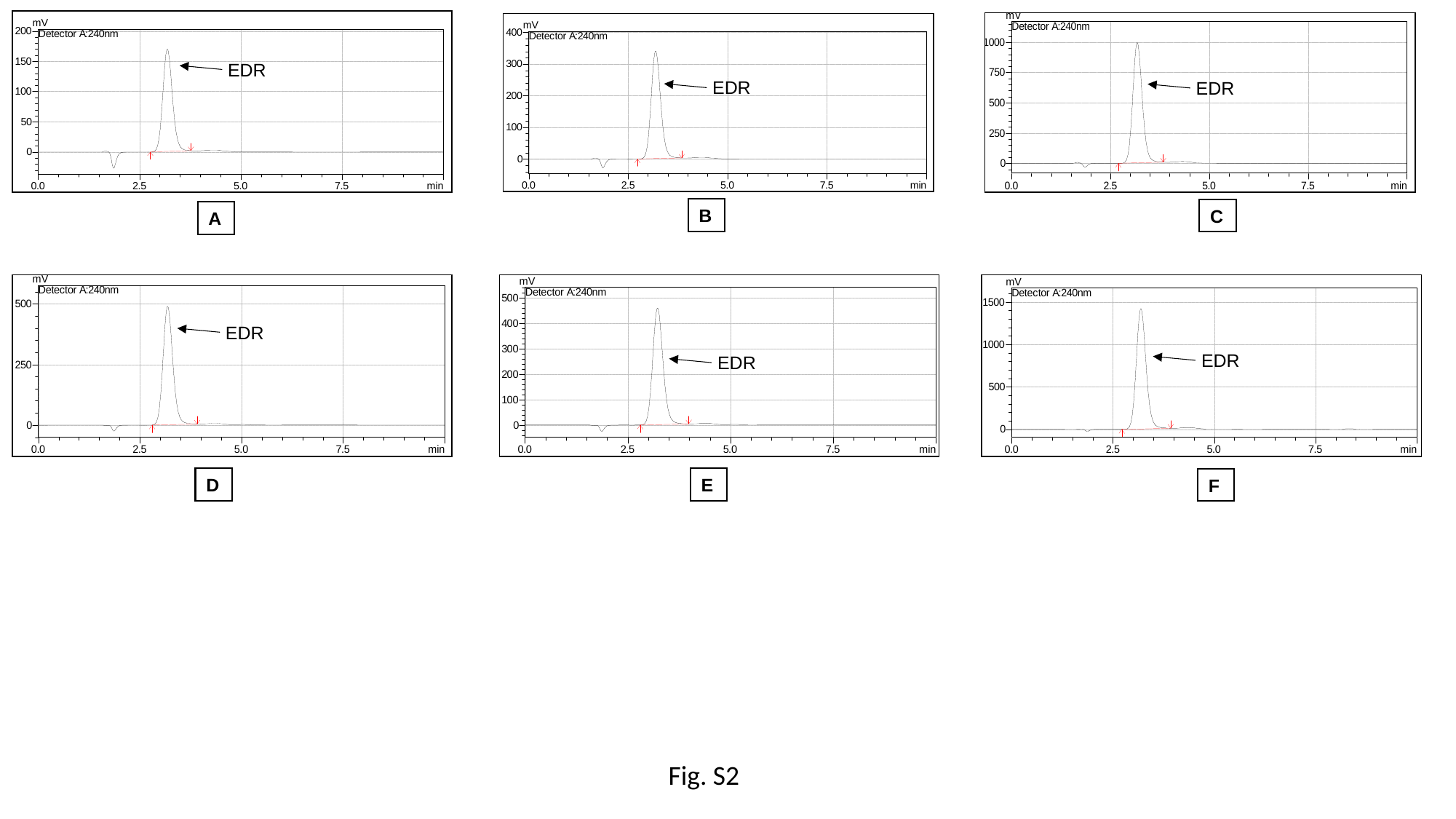

EDR
EDR
EDR
B
C
A
EDR
EDR
EDR
E
D
F
Fig. S2

## Slide 3
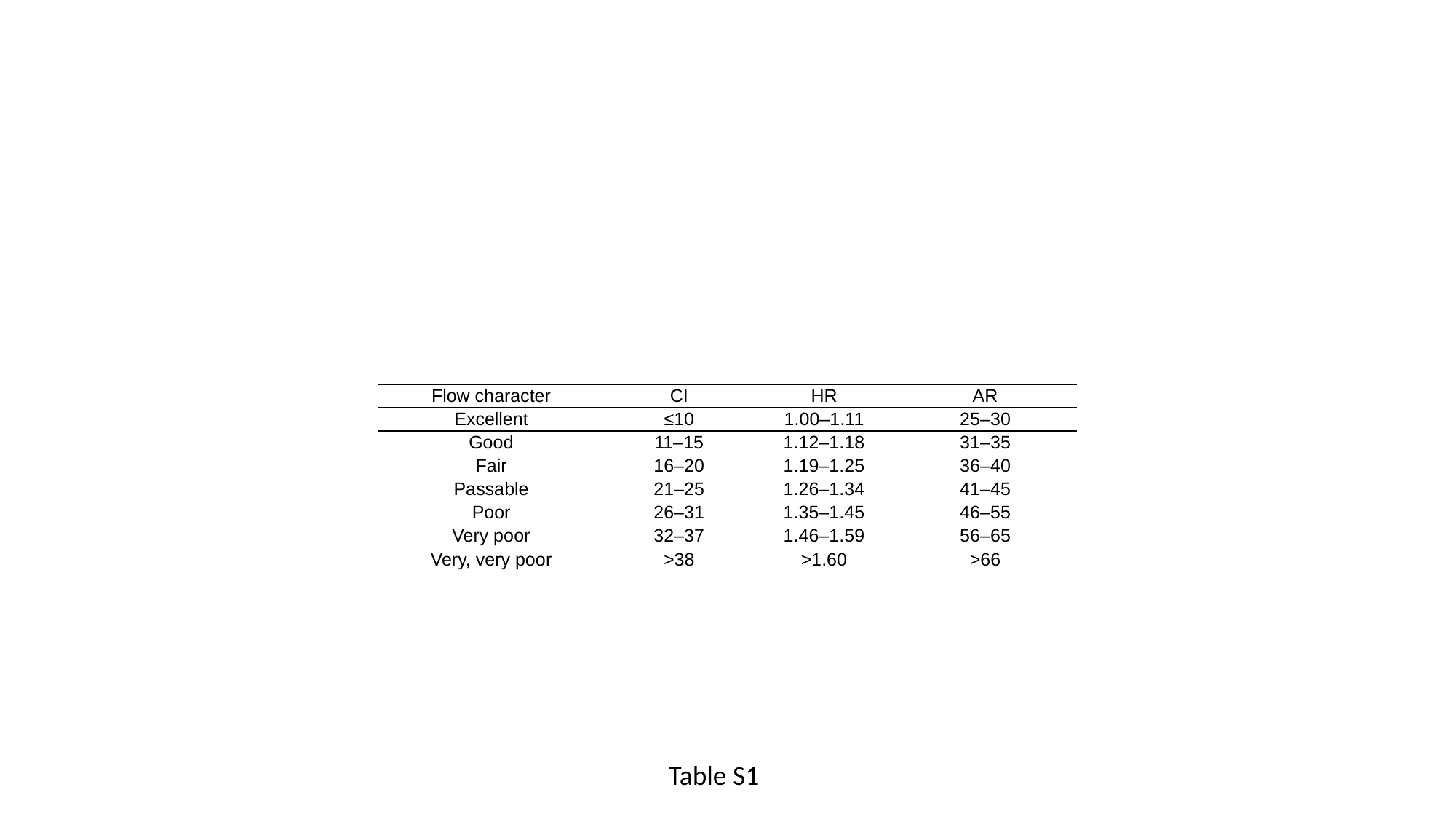

| Flow character | CI | HR | AR |
| --- | --- | --- | --- |
| Excellent | ≤10 | 1.00–1.11 | 25–30 |
| Good | 11–15 | 1.12–1.18 | 31–35 |
| Fair | 16–20 | 1.19–1.25 | 36–40 |
| Passable | 21–25 | 1.26–1.34 | 41–45 |
| Poor | 26–31 | 1.35–1.45 | 46–55 |
| Very poor | 32–37 | 1.46–1.59 | 56–65 |
| Very, very poor | >38 | >1.60 | >66 |
Table S1

## Slide 4
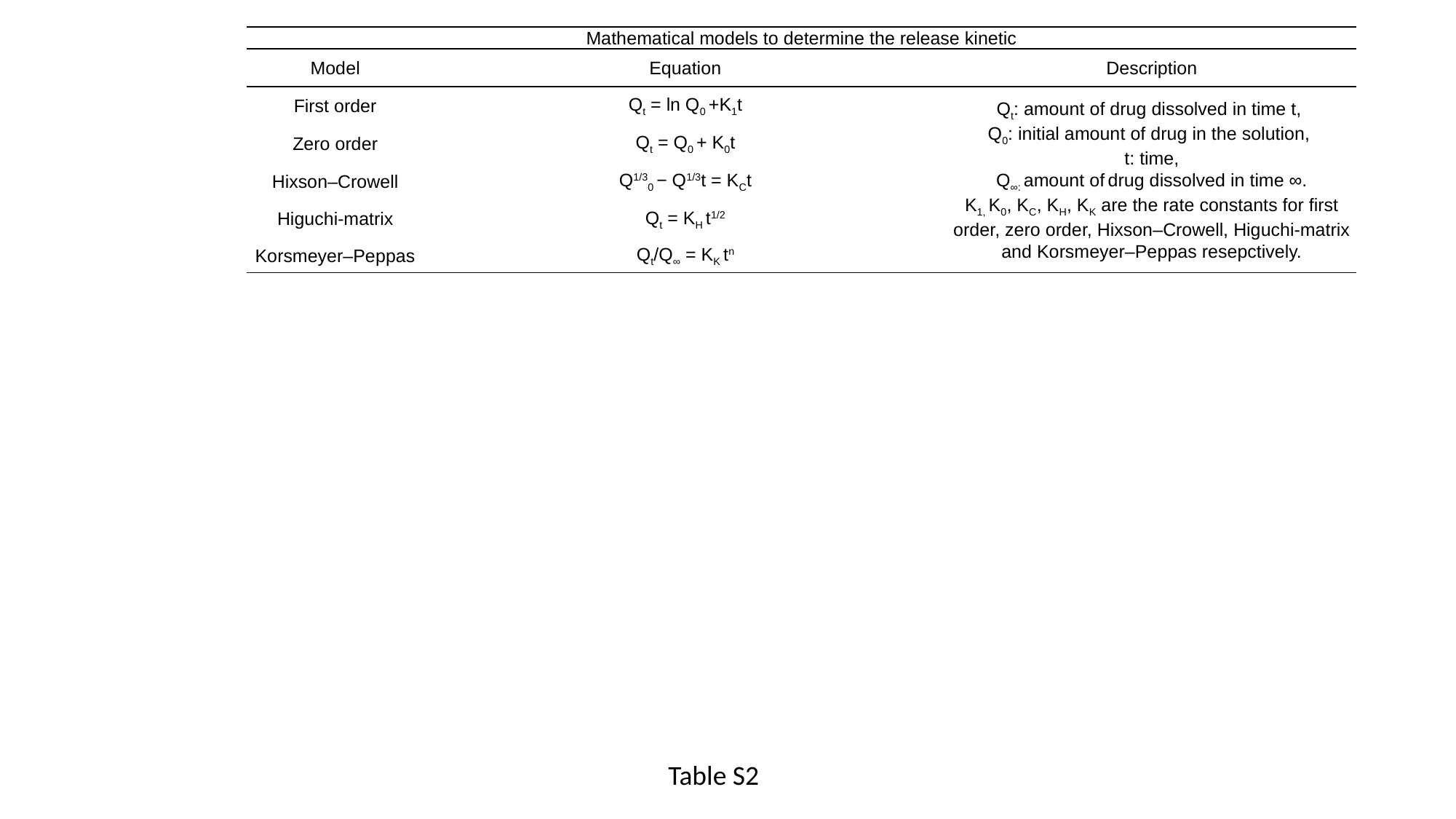

| Mathematical models to determine the release kinetic | | |
| --- | --- | --- |
| Model | Equation | Description |
| First order | Qt = ln Q0 +K1t | Qt: amount of drug dissolved in time t, Q0: initial amount of drug in the solution, t: time, Q∞: amount of drug dissolved in time ∞. K1, K0, KC, KH, KK are the rate constants for first order, zero order, Hixson–Crowell, Higuchi-matrix and Korsmeyer–Peppas resepctively. |
| Zero order | Qt = Q0 + K0t | |
| Hixson–Crowell | Q1/30 − Q1/3t = KCt | |
| Higuchi-matrix | Qt = KH t1/2 | |
| Korsmeyer–Peppas | Qt/Q∞ = KK tn | |
Table S2

## Slide 5
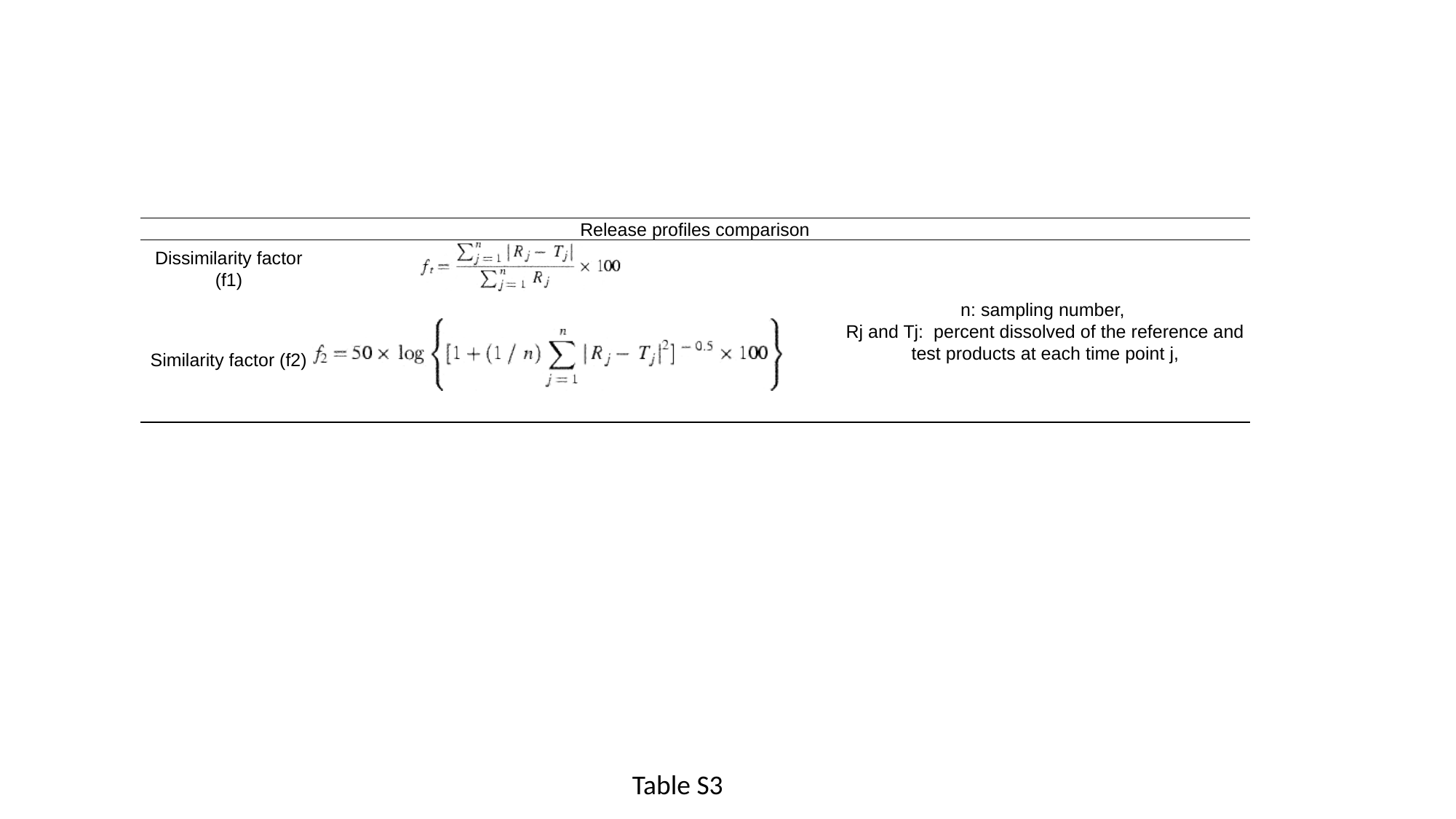

| Release profiles comparison | | |
| --- | --- | --- |
| Dissimilarity factor (f1) | | n: sampling number, Rj and Tj: percent dissolved of the reference and test products at each time point j, |
| Similarity factor (f2) | | |
Table S3

## Slide 6
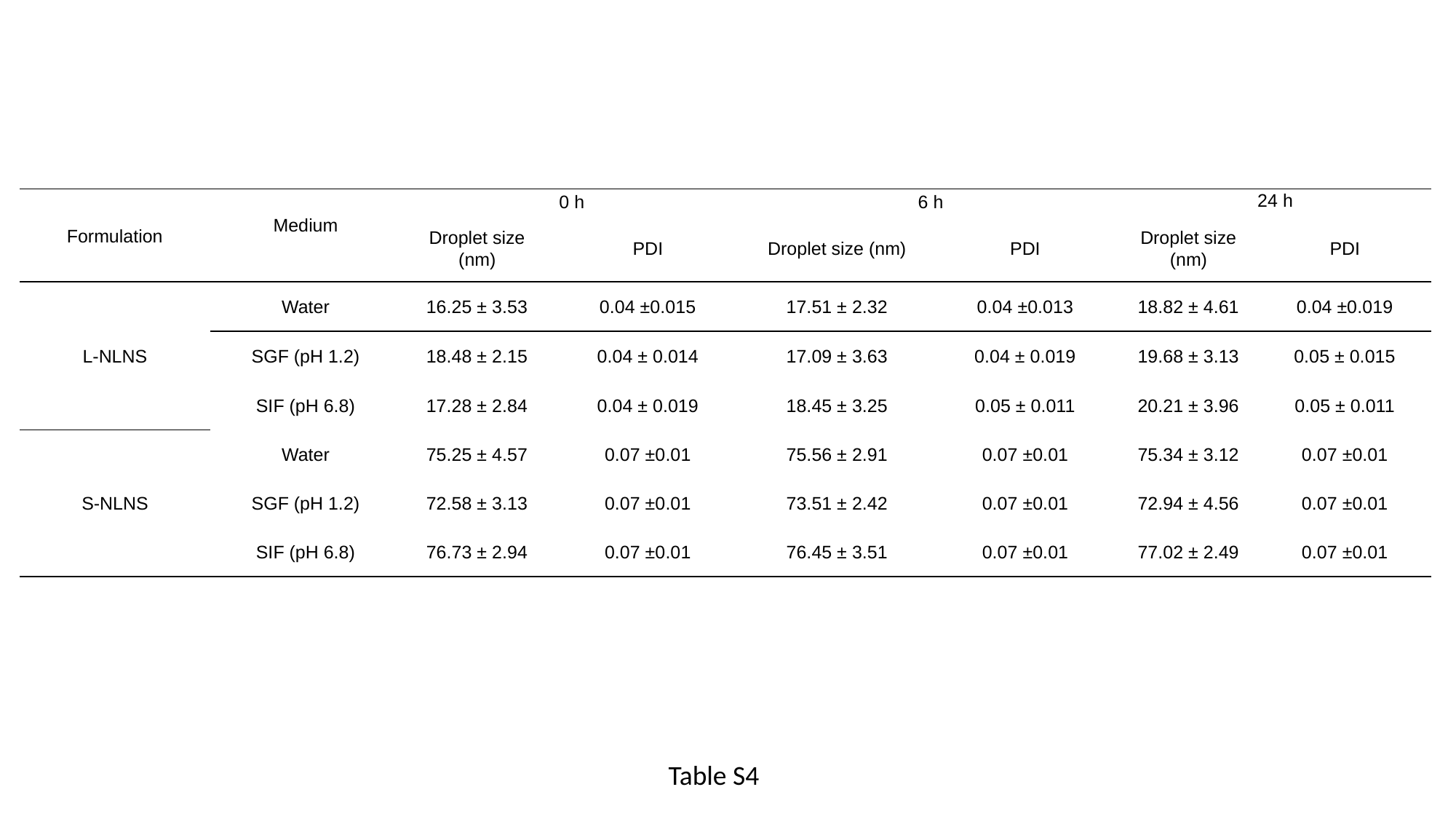

| Formulation | Medium | 0 h | | 6 h | | 24 h | |
| --- | --- | --- | --- | --- | --- | --- | --- |
| | | Droplet size (nm) | PDI | Droplet size (nm) | PDI | Droplet size (nm) | PDI |
| L-NLNS | Water | 16.25 ± 3.53 | 0.04 ±0.015 | 17.51 ± 2.32 | 0.04 ±0.013 | 18.82 ± 4.61 | 0.04 ±0.019 |
| | SGF (pH 1.2) | 18.48 ± 2.15 | 0.04 ± 0.014 | 17.09 ± 3.63 | 0.04 ± 0.019 | 19.68 ± 3.13 | 0.05 ± 0.015 |
| | SIF (pH 6.8) | 17.28 ± 2.84 | 0.04 ± 0.019 | 18.45 ± 3.25 | 0.05 ± 0.011 | 20.21 ± 3.96 | 0.05 ± 0.011 |
| S-NLNS | Water | 75.25 ± 4.57 | 0.07 ±0.01 | 75.56 ± 2.91 | 0.07 ±0.01 | 75.34 ± 3.12 | 0.07 ±0.01 |
| | SGF (pH 1.2) | 72.58 ± 3.13 | 0.07 ±0.01 | 73.51 ± 2.42 | 0.07 ±0.01 | 72.94 ± 4.56 | 0.07 ±0.01 |
| | SIF (pH 6.8) | 76.73 ± 2.94 | 0.07 ±0.01 | 76.45 ± 3.51 | 0.07 ±0.01 | 77.02 ± 2.49 | 0.07 ±0.01 |
Table S4

## Slide 7
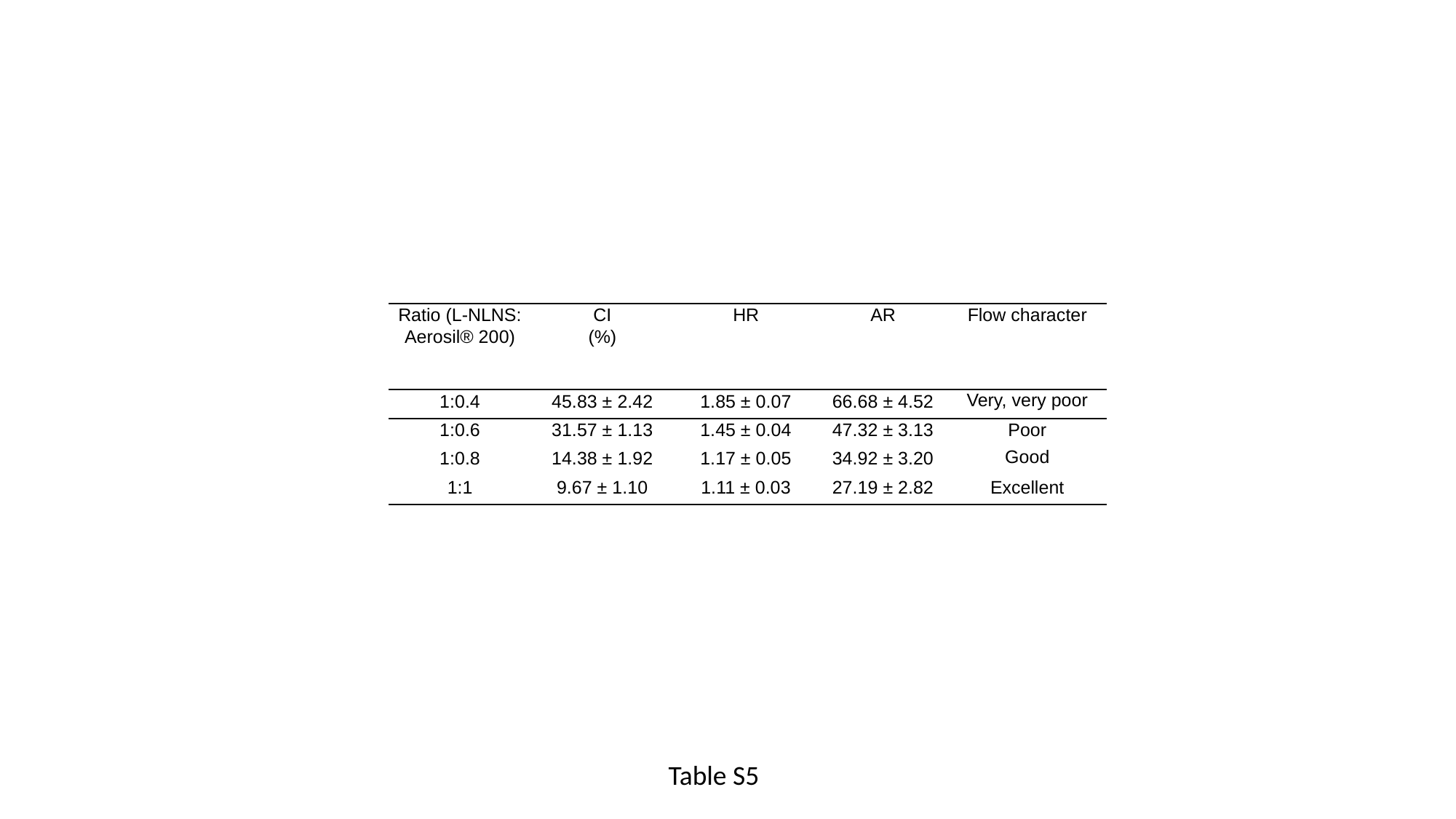

| Ratio (L-NLNS: Aerosil® 200) | CI (%) | HR | AR | Flow character |
| --- | --- | --- | --- | --- |
| 1:0.4 | 45.83 ± 2.42 | 1.85 ± 0.07 | 66.68 ± 4.52 | Very, very poor |
| 1:0.6 | 31.57 ± 1.13 | 1.45 ± 0.04 | 47.32 ± 3.13 | Poor |
| 1:0.8 | 14.38 ± 1.92 | 1.17 ± 0.05 | 34.92 ± 3.20 | Good |
| 1:1 | 9.67 ± 1.10 | 1.11 ± 0.03 | 27.19 ± 2.82 | Excellent |
Table S5

## Slide 8
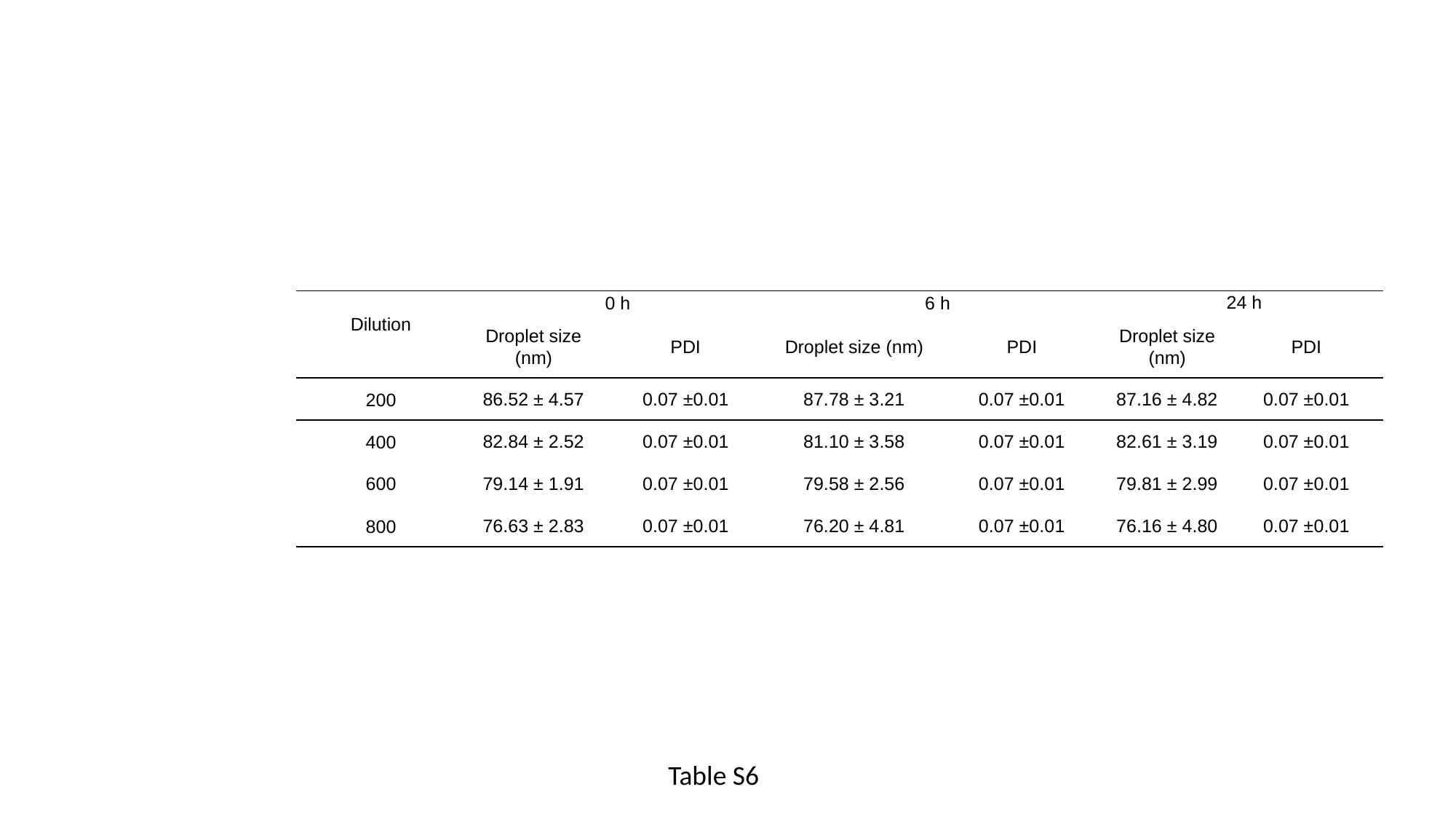

| Dilution | 0 h | | 6 h | | 24 h | |
| --- | --- | --- | --- | --- | --- | --- |
| | Droplet size (nm) | PDI | Droplet size (nm) | PDI | Droplet size (nm) | PDI |
| 200 | 86.52 ± 4.57 | 0.07 ±0.01 | 87.78 ± 3.21 | 0.07 ±0.01 | 87.16 ± 4.82 | 0.07 ±0.01 |
| 400 | 82.84 ± 2.52 | 0.07 ±0.01 | 81.10 ± 3.58 | 0.07 ±0.01 | 82.61 ± 3.19 | 0.07 ±0.01 |
| 600 | 79.14 ± 1.91 | 0.07 ±0.01 | 79.58 ± 2.56 | 0.07 ±0.01 | 79.81 ± 2.99 | 0.07 ±0.01 |
| 800 | 76.63 ± 2.83 | 0.07 ±0.01 | 76.20 ± 4.81 | 0.07 ±0.01 | 76.16 ± 4.80 | 0.07 ±0.01 |
Table S6

## Slide 9
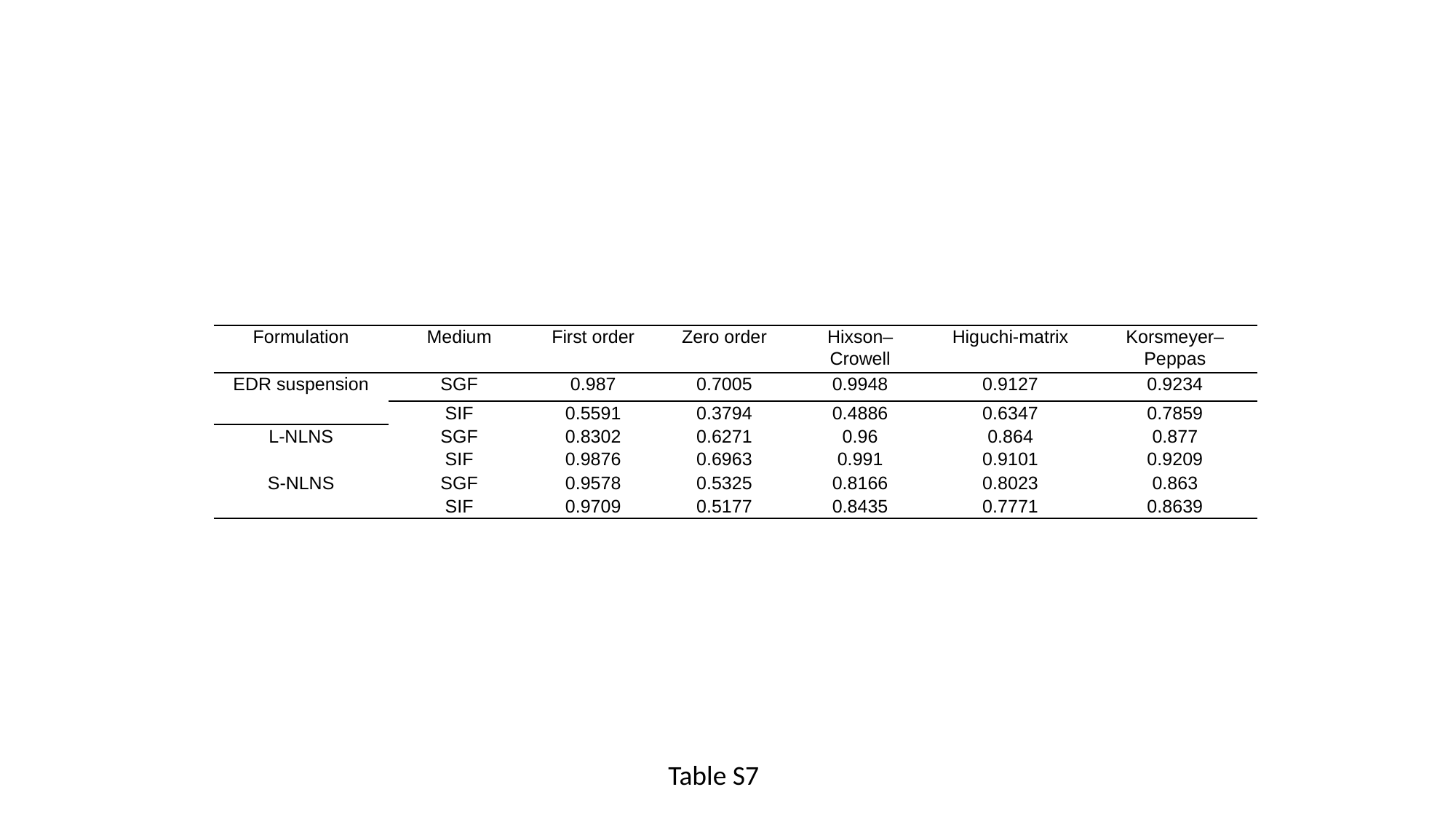

| Formulation | Medium | First order | Zero order | Hixson–Crowell | Higuchi-matrix | Korsmeyer–Peppas |
| --- | --- | --- | --- | --- | --- | --- |
| EDR suspension | SGF | 0.987 | 0.7005 | 0.9948 | 0.9127 | 0.9234 |
| | SIF | 0.5591 | 0.3794 | 0.4886 | 0.6347 | 0.7859 |
| L-NLNS | SGF | 0.8302 | 0.6271 | 0.96 | 0.864 | 0.877 |
| | SIF | 0.9876 | 0.6963 | 0.991 | 0.9101 | 0.9209 |
| S-NLNS | SGF | 0.9578 | 0.5325 | 0.8166 | 0.8023 | 0.863 |
| | SIF | 0.9709 | 0.5177 | 0.8435 | 0.7771 | 0.8639 |
Table S7
